# Evaluation of the Phenotypic Repeatability of Canopy Temperature in Wheat Using Continuous-Terrestrial and Airborne Measurements

**DOI:** 10.3389/fpls.2019.00875

**Published:** 2019-07-09

**Authors:** David M. Deery, Greg J. Rebetzke, Jose A. Jimenez-Berni, William D. Bovill, Richard A. James, Anthony G. Condon, Robert T. Furbank, Scott C. Chapman, Ralph A. Fischer

**Affiliations:** ^1^CSIRO Agriculture and Food, Canberra, ACT, Australia; ^2^ARC Centre of Excellence for Translational Photosynthesis, Australian National University, Canberra, ACT, Australia; ^3^CSIRO Agriculture and Food, Brisbane, QLD, Australia; ^4^School of Food and Agricultural Sciences, The University of Queensland, St. Lucia, QLD, Australia

**Keywords:** field experiments, proximal sensing, remote sensing, data processing, field phenotyping, foliage temperature

## Abstract

Infrared canopy temperature (CT) is a well-established surrogate measure of stomatal conductance. There is ample evidence showing that genotypic variation in stomatal conductance is associated with grain yield in wheat. Our goal was to determine when CT repeatability is greatest (throughout the season and within the day) to guide CT deployment for research and wheat breeding. CT was measured continuously with ArduCrop wireless infrared thermometers from post-tillering to physiological maturity, and with airborne thermography on cloudless days from manned helicopter at multiple times before and after flowering. Our experiments in wheat, across two years contrasting for water availability, showed that repeatability for CT was greatest later in the season, during grain-filling, and usually in the afternoon. This was supported by the observation that repeatability for ArduCrop, and more so for airborne CT, was significantly associated (*P* < 0.0001) with calculated clear-sky solar radiation and to a lesser degree, vapor pressure deficit. Adding vapor pressure deficit to a model comprising either clear-sky solar radiation or its determinants, day-of-year and hour-of-day, made little to no improvement to the coefficient of determination. Phenotypic correlations for airborne CT afternoon sampling events were consistently high between events in the same year, more so for the year when soil water was plentiful (*r* = 0.7 to 0.9) than the year where soil water was limiting (*r* = 0.4 to 0.9). Phenotypic correlations for afternoon airborne CT were moderate between years contrasting in soil water availability (*r* = 0.1 to 0.5) and notably greater on two separate days following irrigation or rain in the drier year, ranging from *r* = 0.39 to 0.53 (*P* < 0.0001) for the midday events. For ArduCrop CT the pattern of phenotypic correlations, within a given year, was similar for both years: phenotypic correlations were higher during the grain-filling months of October and November and for hours-of-day from 11 onwards. The lowest correlations comprised events from hours-of-day 8 and 9 across all months. The capacity for the airborne method to instantaneously sample CT on hundreds of plots is more suited to large field experiments than the static ArduCrop sensors which measure CT continuously on a single experimental plot at any given time. Our findings provide promising support for the reliable deployment of CT phenotyping for research and wheat breeding, whereby the high repeatability and high phenotypic correlations between afternoon sampling events during grain-filling could enable reliable screening of germplasm from only one or two sampling events.

## 1. Introduction

Canopy temperature (CT) has been used in field phenotyping of crops since the 1960s (e.g., Fuchs and Tanner, 1966). The use of CT is based on the fact that plant surfaces (e.g., leaves) are cooled by evaporation, so that temperatures decrease in proportion to the evaporation rate. In this way, cooler CT is related to stomatal opening and higher transpiration rates while in contrast, stomatal closure and a reduction in transpiration rate manifests as a warmer CT. Thus, CT can be used as a surrogate measure of stomatal traits including stomatal conductance, stomatal aperture or leaf porosity and indirectly, photosynthetic rate (Blum et al., 1982; Smith et al., 1988; Amani et al., 1996; Fischer et al., 1998; Jones, 2004; Leinonen et al., 2006; Jones and Vaughan, 2010; Maes and Steppe, 2012). The latter arises because of the dependence of photosynthetic gas exchange on stomatal conductance and the two are often highly correlated. However, CT could be insensitive to non-stomatal regulation of photosynthesis. The relationship between stomatal conductance and yield potential in C3 crops over the last 50 years was recently highlighted in a review (Roche, 2015). Further, under yield potential conditions, cooler CT has been associated with genetic gains in wheat yield (Aisawi et al., 2015), and higher stomatal conductance and maximum photosynthetic rate in the CIMMYT wheat breeding program (Fischer et al., 1998). Likewise, cooler CT has been associated with grain yield under warm irrigated conditions in Mexico (Reynolds et al., 1994; Amani et al., 1996; Ayeneh et al., 2002; Rutkoski et al., 2016) and in Australian environments (Rattey et al., 2011; Rebetzke et al., 2013b). Similarly, in water-limited environments, cooler CT has been associated with increased wheat yield (Blum et al., 1989; Rashid et al., 1999; Olivares-Villegas et al., 2007). While lower CT may be linked directly to yield via greater stomatal conductance under yield potential conditions, another possibility arises under water limitation: cooler CT has been associated with increased rooting depth (Reynolds et al., 2007), and greater water use and yield (Lopes and Reynolds, 2010) when measured during grain-filling.

The use of airborne thermography in field experiments has greatly increased the repeatability of CT. Previous hand-held CT heritability estimates were low of the order of 0.1 to 0.3 (e.g., Rebetzke et al., 2003, 2013b; Pask et al., 2012). In contrast, using airborne CT, Deery et al. (2016) reported broad-sense heritabilities typically >0.50 and as high as 0.79. In a study comprising five environments and several hundred breeding lines, broad-sense heritabilities for airborne CT, estimated on a single-plot and line-mean basis, were high ranging from 0.56 to 0.96 (Rutkoski et al., 2016). To the best of our knowledge, no study has reported estimates of CT repeatability from ArduCrop CT.

The greater heritability now achievable through airborne thermography (Deery et al., 2016; Rutkoski et al., 2016), together with the demonstrable association between stomatal conductance and grain yield improvement (Roche, 2015), highlights the potential for deployment of CT within a breeding program as an indirect surrogate for grain yield. The value of CT deployment is likely to be greatest in early generations (Rebetzke et al., 2002; Fischer and Rebetzke, 2018), on unreplicated rows or small plots where reliable yield measures are unattainable (Rebetzke et al., 2014). Further opportunities include improving the heritability estimate of grain yield by using CT measurements to improve spatial and site characterization for variation in soil water, and subsoil constraints including root disease (Araus et al., 2018).

In order for CT to be effectively utilized within a wheat breeding program, a greater understanding is required of: (1) the optimal period of the season (e.g., before and or after flowering) and the optimal time during the day to measure CT; (2) the benefits of aerial vs static CT measurements; and (3) the number of measurements required in a given year to appropriately characterize the germplasm. We address these issues in this paper through the use of continuous terrestrial and regular airborne CT measurements to evaluate the repeatability of CT at discrete time points and the phenotypic correlation across and between two seasons contrasting in soil water availability.

## 2. Materials and Methods

### 2.1. Field Experiments

A field experiment containing wheat genotypes contrasting for canopy architecture was grown in two successive years at the Managed Environment Facility (MEF) (Rebetzke et al., 2013a), located at Yanco (34.62°S, 146.43°E, elevation 164 m) in South-eastern Australia. The soil at the Yanco MEF is classified as chromosol and has a clay-loam texture (Isbell, 1996). The experiment was sown on 23rd May in 2016 and 29th May in 2017 following canola or field pea break-crops and then managed with adequate nutrition and chemical controls as required for pest, weed and leaf diseases.

The experiment comprised 400 and 192 experimental plots, in 2016 and 2017, respectively, of size 2 × 6 m containing seven rows of 25 cm spacing (orientated North - South), sowing density of 200 seeds per mʦ2 and paths between plots of *ca*. 0.4 m. The germplasm represented a series of near-isogenic wheat lines varying for a range of agronomic traits including plant height, tiller number, plant development and canopy erectness. In 2016, 106 genotypes were sown into a partial-replicate design experiment with the genotype replication averaging 3.8 and ranging from one to five. In 2017, 99 genotypes were sown into a partial-replicate design experiment comprising 192 plots with the genotype replication averaging 1.9 and ranging from one to two. Ninety-eight of the 99 genotypes grown in 2017 were also grown in 2016. The dimensions of the experiment were 50 × 110 m in 2016 and 25 × 110 m in 2017.

In 2016, 670 mm of rainfall was recorded at the site between 1-Jan-2016 and when the crop reached physiological maturity (1-Dec-2016). Of this, 191 mm was recorded prior to sowing and the remaining 479 mm between sowing and harvest. In 2017, 201 mm of rainfall was recorded at the site between 1-Jan-2017 and physiological maturity (1-Dec-2017). Of this, 90 mm was recorded prior to sowing and the remaining 111 mm between sowing and physiological maturity. Due to the limited rainfall in 2017, a total of 186 mm of sprinkler irrigation was applied on seven separate days throughout the season, with amounts ranging from 15 to 37 mm. Thus, rainfall and irrigation totalled 387 mm in 2017, 283 mm less than the total rainfall in 2016.

In 2016, for 90% of the lines, the flowering growth stage ranged from 22-Sept-16 to 13-Oct-16 (122 to 143 days after sowing, respectively) and the median flowering date was 28-Sept-16 (128 days after sowing). In 2017, for 90% of the lines, the flowering growth stage ranged from 25-Sept-17 to 10-Oct-17 (119 to 134 days after sowing, respectively) and the median flowering date was 3-Oct-17 (127 days after sowing). Therefore, results are presented according to the following growth stages: early-veg, early vegetative growth stage (August); late-veg, late vegetative growth stage (September); early-gf, early grain-filling growth stage (October); late-gf, late grain-filling growth stage (November).

### 2.2. Weather Measurements

For 2016 and 2017, the following weather variables were obtained from the Bureau of Meteorology (http://www.bom.gov.au) weather station located at the experiment site (station number 074037): air temperature (°C); average and maximum wind speed (*km*.*hr*^−1^); wind direction (°); and vapor pressure deficit (VPD) (*Pa*). These variables were measured at 60 s frequency. The clear-sky solar radiation, *R*_*so*_ (*W*.*m*^−2^), was calculated as 75% of the extraterrestrial solar radiation, whereby the latter was calculated hourly from the day-of-year and latitude (Allan et al., 1998) for both 2016 and 2017. In 2016 and 2017, solar radiation, *R*_*s*_ (*W*.*m*^−2^), was measured hourly at Griffith NSW (*ca*. 60 km north-west from the experiment site).

### 2.3. Continuous Canopy Temperature Measurements

**Figure 1:**
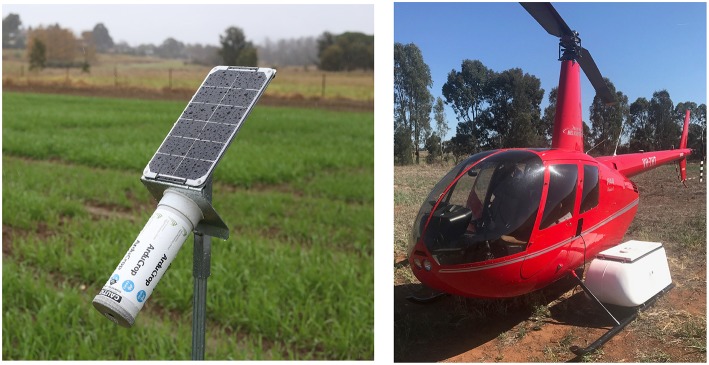
ArduCrop wireless infra-red canopy temperature sensor **(Left)** and manned helicopter for airborne canopy temperature **(Right)** comprising white cargo pod mounted on skid of helicopter with thermal camera inside.

Continuous CT measurements were made with the ArduCrop wireless canopy temperature system described previously (Rebetzke et al., 2016; Jones et al., 2018) (Figure 1). The ArduCrop system comprises wireless infrared temperature sensors, similar in design to that described by O'Shaughnessy et al. (2011a,b), with an infrared thermometer sensor (MLX90614-BCF from Melexis, Ypres, Belgium), for which the technical specifications are: 10° field of view; resolution of 0.02°C; and accuracy of ± 0.5°C from 0 to 50°C. This specification was checked for each ArduCrop sensor before and after deployment with a Landcal P80P black body radiation source (Land Instruments, Leicester, United Kingdom). Temperature data were recorded at 1 s intervals on an Arduino microcontroller and 60 s averages radio transmitted to a field base station. The base station sent data via the mobile phone network every 15 min to the SensorDB website [http://sensordb.csiro.au, see Salehi et al. (2015)] for real-time data access and preliminary visualization through a web portal. The ArduCrop sensors were height adjustable to maintain a consistent height for all plots above the crop canopy throughout the growing season of *ca*. 0.5 m. The ArduCrop viewing angle was 45° facing toward the canopy. Thus, each ArduCrop sensor collected data from an elliptical field of view *ca*. 0.2 m long by *ca*. 0.1 m wide. Each ArduCrop sensor was positioned to view the canopy at ca. 45° angle to the individual rows, and thereby reduce the likelihood of viewing background soil, and facing approximately north-northwest (in Southern Hemisphere) to avoid the warmer, sunlit side of the canopy (see Jones, 2002).

In 2016, 113 ArduCrop sensors in total were deployed from 11-Aug-2016 until 24-Nov-2016, across 43 randomly sampled genotypes on 84 of the 400 experimental plots. Of the 84 experimental plots containing ArduCrop sensors, 55 plots contained one ArduCrop sensor and 29 plots contained two paired ArduCrop sensors, referred to as duplicate-ArduCrop-plots. The duplicate-ArduCrop-plots enabled testing whether the additional ArduCrop sensor improved the estimate of CT. The plot level replication per genotype ranged from one to four and averaged 1.95.

In 2017, 96 ArduCrop sensors were deployed from 24-Aug-2017 until 27-Nov-2017, across 49 randomly sampled genotypes, 20 of which were also used in 2016. ArduCrop replication per genotype in 2017 ranged from one to two and averaged 1.96. Duplicate-ArduCrop-plots were not used in 2017.

### 2.4. Airborne Canopy Temperature Measurements

As previously described (Deery et al., 2016), thermal images were acquired using a thermal infrared camera (FLIRʦ® SC645, FLIR Systems, Oregon, USA, for which the technical specifications are: ±2°C or ±2% of reading; < 0.05°C pixel sensitivity; 640x480 pixels; 0.7 kg without lens; 13.1 mm lens). The camera was mounted in a commercially-available helicopter cargo pod (R44 Helipod II Slim Line Top Loader, Simplex Aerospace, Oregon, USA) and fitted to a Robinson R44 Raven helicopter (Figure 1). Images for a given event were collected in a single pass and typically acquired at a height of 120 m above-ground-level and at a flight velocity of 25 to 35 knots (45 to 65 km/h). The camera was mounted to provide a nadir view (pointing straight down), such that the angle of view for a given image spread from vertical to 15-20° at the image edges. Thus, images for a given event were acquired in <10 s for the experiments described above.

Measurements of airborne CT occurred on six and eight separate days in 2016 and 2017, respectively. On a given day, measurements generally occurred hourly starting at 09:00 and finishing at 15:00. Herein an airborne CT measurement at a given date and time is referred to as an event.

### 2.5. Data Processing

The Python 3.5 software language (Python Software Foundation, https://www.python.org) was used for data processing [pandas and NumPy modules (Jones et al., 2001)]. All data is reported in local time for the experiment site, namely Australian Eastern Standard Time (UTC/GMT +10 h) and, during daylight savings time, Australian Eastern Daylight Time (UTC/GMT +11 h). Note that daylight saving time commenced at 02:00 on 1-Oct-2016 and 02:00 on 1-Oct-2017.

#### 2.5.1. Weather Data

Hourly means were calculated from the 60 s weather data using the pandas module in Python 3.5 [method: resample(“H”).mean()], whereby an hourly mean computed for 12:00 comprises values from 12:00 to 12:59 inclusive.

#### 2.5.2. ArduCrop Canopy Temperature Data

For each ArduCrop sensor, temperature data <–30.0°C and >50.0°C was attributed to the ArduCrop sensor inadvertently viewing the sky or soil and was therefore discarded, prior to the calculation of hourly mean. ArduCrop sensor data was also discarded on days when rainfall and irrigation occurred. Then for each day, hourly mean data between the times of 08:00 and 16:00, inclusive, were calculated for later analysis (refer section 2.6) using same method described above for the weather data.

#### 2.5.3. Airborne Canopy Temperature Data

Thermal images were processed using a previously described method (Deery et al., 2016), whereby the CT for each individual plot was extracted for later analysis. Custom developed software works on a frame-by-frame basis extracting data from the raw images, whereby the user navigates through the image stack to ensure that each plot in the experiment has been sampled. For each experimental plot, a rectangle was defined within a surrounding buffer, and the CT pixels extracted from within the plot rectangle. From the resultant pixels within each plot, a mean CT for a given plot was calculated for later analysis (refer section 2.6).

### 2.6. Statistical Analysis

Hourly ArduCrop and airborne CT data were analyzed after first checking for residual normality and error variance homogeneity at each date-by-time sampling event. Each event was analyzed separately using the SpATS package (Rodríguez-Álvarez et al., 2018) (available from CRAN: https://cran.r-project.org/package=SpATS) in the R programming language (http://www.r-project.org). Spatial effects were modeled on a row and column basis by specifying the separation of anisotropic penalties (SAP) algorithm, with the number of segments set to the respective number of rows and columns from the experimental design. For the 2016 ArduCrop data, where for the purpose of the analysis the duplicate ArduCrops in the duplicate-ArduCrop-plots were treated as internal replicates (or pseudo-replicates), the following factors were modeled as random effects: genotype, row, column and the internal ArduCrop replicate. For the 2016 airborne, 2017 ArduCrop and 2017 airborne CT data, the following factors were modeled as random effects: genotype, row and column. Repeatability (ρ), sometimes called broad-sense heritability (Falconer and Mackay, 1996; Holland et al., 2003; Piepho and Möhring, 2007), was then estimated using relevant variance components, namely: $\rho =\frac{\sigma^{2}{\;}_{g}}{\left(\sigma^{2}{\;}_{g}+\frac{\sigma^{2}{\;}_{\epsilon }}{nrep}\right)}\;$. Where $\sigma^{2}{\;}_{g}$ and $\sigma^{2}{\;}_{\epsilon }$ are the genotypic and residual variances, respectively, and *nrep* is the number of genotype replicates in the experiment. The best linear unbiased predictors of genotype effects (BLUPs) and standard errors (BLUP SEs) were predicted from a fitted SpATS object. Phenotypic correlations were estimated between BLUPs using Pearson correlation analysis with the pandas module in Python 3.5 and statistically significant associations denoted: ^****^P < 0.0001; ^***^P < 0.001; ^**^P < 0.01; ^*^P < 0.05. The scipy module (Jones et al., 2001) in Python 3.5 was used to estimate linear least-squares regression and the coefficient of determination (*R*^2^) between variables.

The association between canopy temperature (CT) repeatability (response variable) and the weather (explanatory) variables most significantly and strongly correlated with repeatability was investigated using ordinary least squares (OLS) regression analysis using the statsmodels Python module (Seabold and Perktold, 2010). Figures were prepared using the matplotlib and seaborn Python modules (Jones et al., 2001). Phenotypic correlations for ArduCrop CT are presented in hierarchically-clustered heatmaps to identify occasions when the phenotypic correlations were greatest. Box plots were used to summarize data according to the following: The box extends from the lower to upper quartile values (Q1 and Q3) of the data, with a line at the median. The whiskers extend from the box by the product of 1.5 and the interquartile range (i.e., Q3+1.5*IQR and Q1-1.5*IQR). The flyer points are data points past the end of the whiskers.

## 3. Results

### 3.1. Summary of Experimental Conditions

The meteorological conditions during the CT measurement period for both years are summarized in Table 1, together with the rainfall, irrigation and the ArduCrop and airborne CT deltas from air temperature (CT minus air temperature). The latter were calculated on two dates (at 13:00 on 5-Oct-16 and 3-Oct-17), using the respective ArduCrop or airborne CT mean of the best linear unbiased predictors of genotype effects, and illustrate the extreme contrast in available soil water between 2016 and 2017, whereby ArduCrop and airborne CT deltas from air temperature were greater in 2017 than 2016. On both dates, ArduCrop CT and airborne CT were warmer than air temperature and airborne CT was warmer than ArduCrop CT. VPDs were greater in 2017 than 2016, indicating that evaporative demand was likely also greater in 2017.

**Table 1:** Experimental conditions during the canopy temperature (CT) measurement period for 2016 and 2017 at Yanco, New South Wales.

		***T*_*min*_**	***T***_***max***_	**VPD**	**Radiation**	**Rain**	**Irrigation**	**ArduCrop CT**	**Airborne CT**
		**(^**°**^C)**	**(^**°**^C)**	**(Pa)**	**(*MJ*.*m*^−2^)**	**(mm)**	**(mm)**	**delta (^**°**^C)**	**delta (^**°**^C)**
**Year**	**Month**								
2016	Aug	5.4	15.7	805	11.9	66	0		
	Sep	7.9	17.3	828	14.9	151	0		
	Oct	8.0	21.4	1548	22.2	35	0	1.0	2.5
	Nov	12.0	28.0	3004	25.3	35	0		
2017	Aug	4.1	15.8	1025	13.3	32	0		
	Sep	7.0	21.6	2033	16.8	1	85		
	Oct	11.4	26.0	2481	23.1	28	64	6.8	10.7
	Nov	15.5	30.0	3426	25.8	21	0		

*Monthly means of daily minimum (T_min_) and maximum air temperature (T_max_); daily maximum vapor pressure deficit (VPD); daily accumulated solar radiation. Rain and irrigation are monthly totals. ArduCrop and Airborne CT deltas from air temperature (CT minus air temperature) were calculated on two dates (at 13:00 on 5-Oct-16 and 3-Oct-17) using the respective ArduCrop or airborne CT mean of the best linear unbiased predictors of genotype effects*.

### 3.2. ArduCrop Internal Replicate

**Figure 2:**
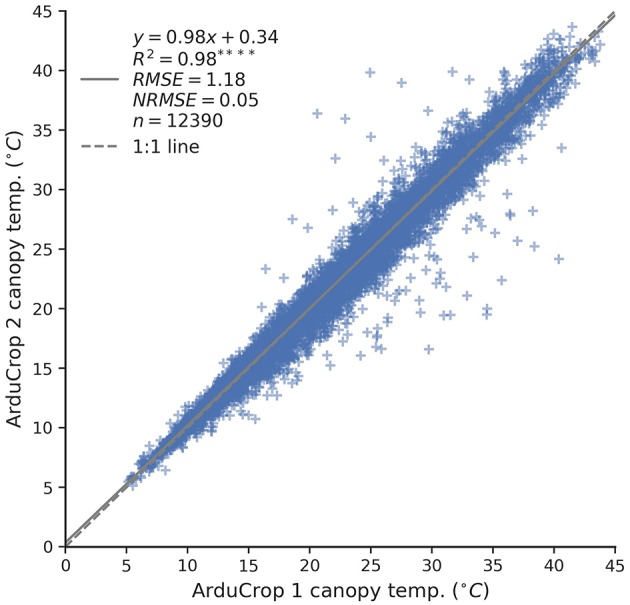
Relationship between canopy temperature (CT) of duplicate ArduCrop sensors, denoted ArduCrop 1 and ArduCrop 2. Scatter plot comprising CT data from all 29 plots with fitted linear regression equation, coefficient of determination (*R*^2^), root mean square error (RMSE), normalized RMSE (NRMSE) and the number of values (*n*). Linear regression analysis for each individual plot is shown in Figure S2 and Table S1.

The internal ArduCrop replicate sensors on the duplicate-ArduCrop-plots were significantly correlated with one another (slope = 0.98, intercept = 0.34°C, *R*^2^ = 0.98, *P* < 0.0001, Figure 2) and the root mean square error (RMSE) was 1.18°C, equating to a normalized RMSE (NRMSE) of 0.05. Linear regression analysis for each individual plot (Figure S2 and Table S1) showed a high degree of linearity between the pairs of ArduCrop sensors (*R*^2^ ≥ 0.96, *P* < 0.0001). The slopes ranged from 0.91 to 1.11, the intercepts ranged from -1.32 to 1.31°C, the RMSE ranged from 0.55 to 1.89°C and the NRMSE was ≤ 0.09. We investigated the size of the variance explained with the addition of an internal ArduCrop replicate on the duplicate-ArduCrop-plots. For the 2016 ArduCrop data, the variance ratios between the internal ArduCrop replication, $\sigma^{2}{\;}_{ArduCrop}$, and $\sigma^{2}{\;}_{\epsilon }$ were typically <0.1 (Figure S1). Specifically, the percentile score denoting when the variance ratios, $\sigma^{2}{\;}_{ArduCrop}$ and $\sigma^{2}{\;}_{\epsilon }$, were <0.1 was 87.

### 3.3. Repeatability of ArduCrop Canopy Temperature

**Figure 3:**
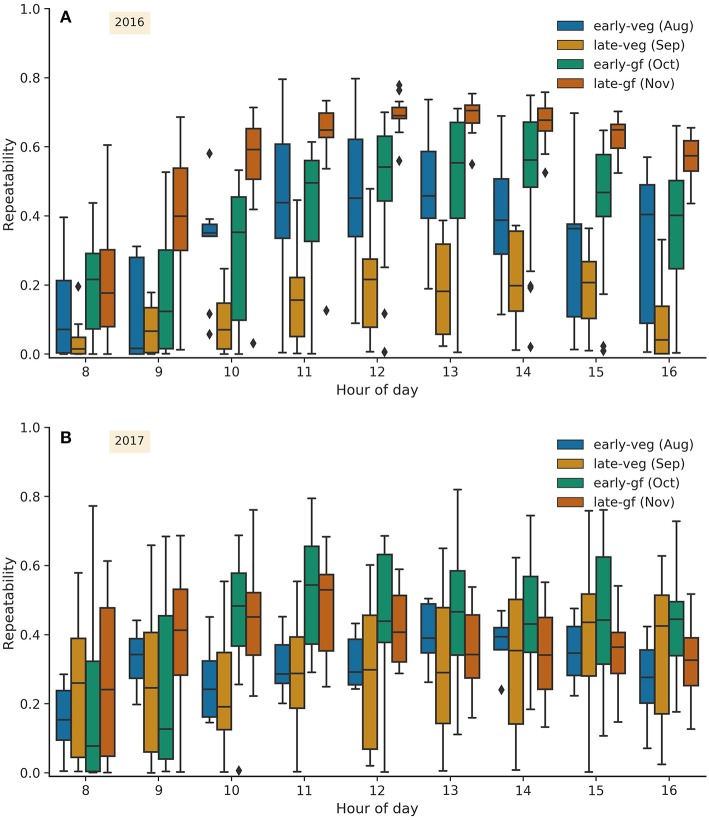
Repeatability estimates for ArduCrop canopy temperature shown as box plots for each hour of the day grouped by growth stage for 2016 **(A)** and 2017 **(B)**. Early-veg, early vegetative; late-veg, late vegetative; early-gf, early grain-filling; late-gf, late grain-filling.

The box plots of repeatability estimates for each hour of the day grouped by growth stage, for 2016 (Figure 3A) and 2017 (Figure 3B), show a similar temporal distribution for both years, although less marked in 2017, whereby repeatability was greater during the grain-filling months of October and November. In 2016, repeatability was highest, and the range lowest, during late-gf (November) and from 11:00 onwards. Estimates of repeatability were also high for the same HoDs in early-gf (October) 2016, however the range was greater cf. late-gf (November) 2016. Repeatability estimates were lowest for HoD 8 and 9, for all months in 2016 and during late-veg (September). For 2017, ArduCrop CT repeatability estimates were highest during early-gf and late-gf (October and November, respectively) for HoD after and including 10, and were also high during late-veg (September) for the afternoon HoDs 15 and 16. For all growth stages at HoD 8 and 9 in 2017, with the exception of late-gf (November) at HoD 9, a large proportion of repeatability values were <*ca*. 0.4. The temporal distributions of repeatability on a weekly basis are shown in Figures S7, S8, for 2016 and 2017, respectively. The frequency distributions of repeatability estimates for ArduCrop CT in 2016 and 2017 are shown in Figure S6. The range in repeatability was large in both years, ranging from 0.0 to 0.80 in 2016 (Figure S6a), and from 0.0 to 0.82 in 2017 (Figure S6b). The median repeatability was similar for both years (0.42 in 2016 and 0.36 in 2017).

### 3.4. Phenotypic Correlations for ArduCrop Canopy Temperature Within Years

**Figure 4:**
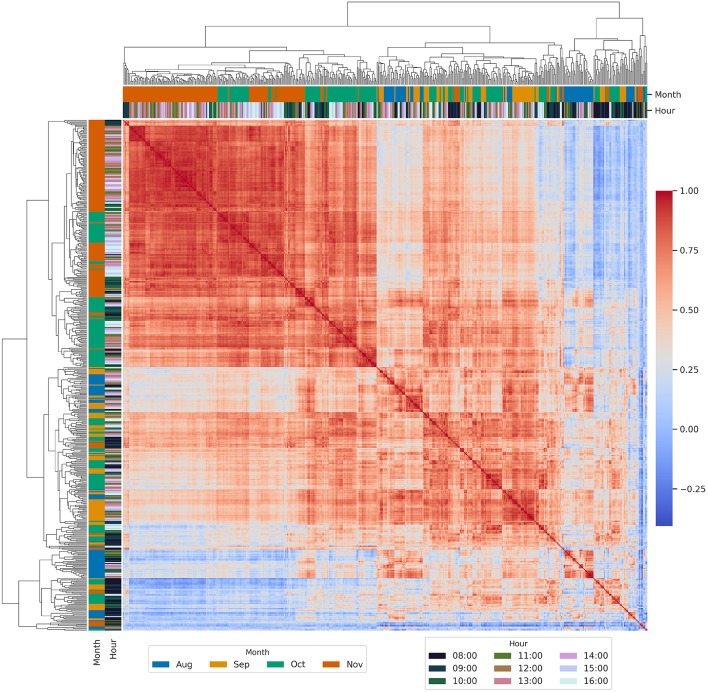
Hierarchically-clustered heatmap of all possible pairwise phenotypic correlations between best linear unbiased predictors of genotype effects (BLUPs) for ArduCrop canopy temperature in 2016. Frequency distributions of the phenotypic correlations are shown in Figure S11.

**Figure 5:**
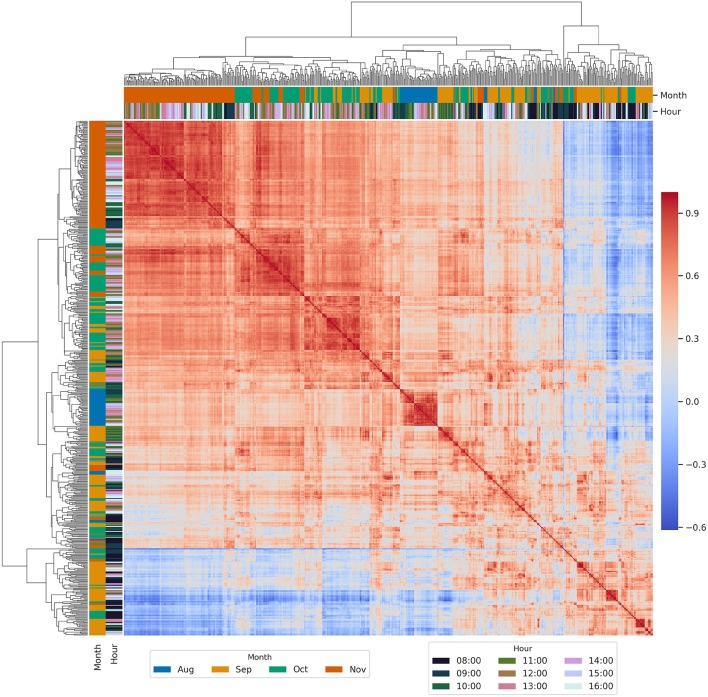
Hierarchically-clustered heatmap of all possible pairwise phenotypic correlations between best linear unbiased predictors of genotype effects (BLUPs) for ArduCrop canopy temperature in 2017. Frequency distributions of the phenotypic correlations are shown in Figure S11.

Hierarchically-clustered heatmaps of all possible pairwise phenotypic correlations between BLUPs are shown for ArduCrop CT in 2016 (Figure 4) and 2017 (Figure 5). The overall pattern of clusters was similar for both years, whereby correlations were higher during the grain-filling months of October and November and for HoDs from 11 onwards. Conversely, the lowest correlations comprised events from HoDs 8 and 9 across all months (clustered at the lower left of the figures).

To investigate the phenotypic correlations between ArduCrop CT BLUPs as a function of repeatability, all possible pairwise phenotypic correlations were estimated for ArduCrop CT BLUPs corresponding to the following, arbitrarily chosen, quantiles of event repeatability: 0.0 to 0.33; 0.33 to 0.66; 0.66 to 1.0 (shown as frequency distributions in Figures S11a–c for 2016 and Figures S11d–f for 2017, respectively). The mean, median and percentiles of phenotypic correlations increased with repeatability quantiles (i.e., 0.0 to 0.33 < 0.33 to 0.66 < 0.66 to 1.0) for both years. For a given quantile distribution (e.g., 0.0 to 0.33 in 2016 cf. 0.0 to 0.33 in 2017 etc.), the mean, median and percentiles of phenotypic correlations were higher for 2016 than 2017.

The frequency distributions of phenotypic correlations for the entire data set of ArduCrop CT BLUPs are shown in Figure S11g for 2016, and Figure S11h for 2017, whereby the mean, median and percentiles of phenotypic correlations were higher for 2016 than 2017. The mean and median was 0.50 and 0.52, in 2016 respectively, and 0.38 and 0.41 in 2017, respectively.

### 3.5. Repeatability of Airborne Canopy Temperature

**Figure 6:**
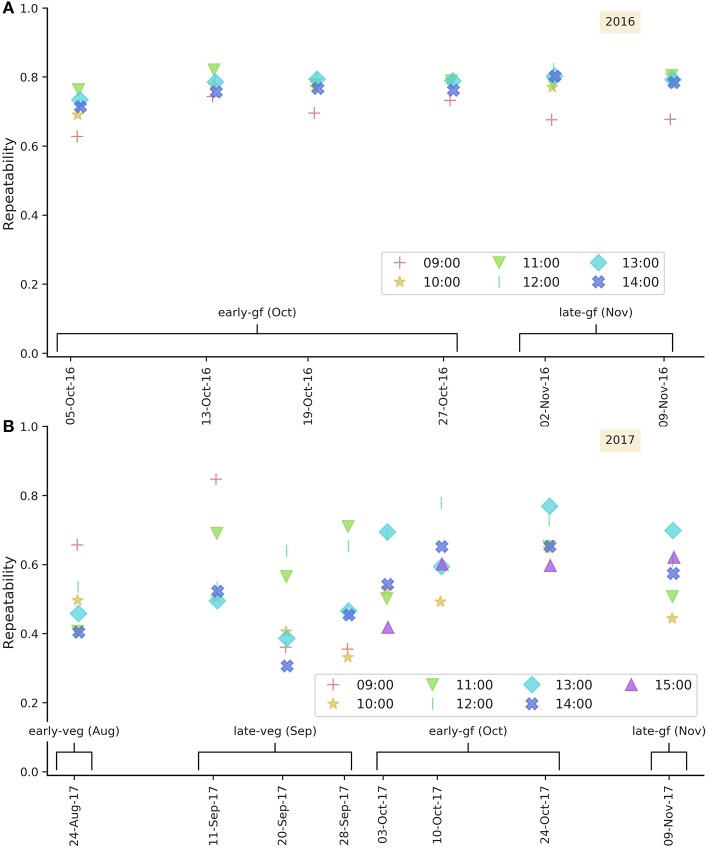
Repeatability estimates for airborne canopy temperature shown as scatter plots for each date in 2016 **(A)** and 2017 **(B)** with hour-of-day denoted as per legend. Growth stage is denoted as follows: early-veg, early vegetative; late-veg, late vegetative; early-gf, early grain-filling; late-gf, late grain-filling.

Repeatability estimates for 2016 and 2017 airborne CT events are shown as scatter plots for each date in Figure 6 (and as frequency distributions in Figure S12). In 2016 all airborne CT events occurred post-flowering during the grain-filling months of October and November. Repeatability estimates in 2016 were typically high, ranging from 0.63 to 0.82 with a mean of 0.76 (Figure S12a). In 2016, the scatter plots of repeatability for each date show that repeatability estimates were typically lower at 09:00, ranging from 0.63 to 0.74 (Figure 6A). In 2017 airborne CT events occurred pre and post-flowering, as denoted on Figure 6B. Repeatability estimates were generally lower in 2017, ranging from 0.31 to 0.85 with a mean of 0.56 (Figure S12b and showed no clear pattern with regards to HoD (Figure 6B). The lowest repeatability estimates occurred during late-veg (20 and 28 September 2017) and, conversely, repeatability estimates were consistently higher for the early-gf and late-gf (October and November, respectively) events. For airborne CT events during early-gf (October) 2017, repeatability tended to increase with HoD until 12, and then decrease with HoD for 13, 14, and 15. Similar patterns were evident for late-gf events (November, one day only), where repeatability increased until HoD 13 before decreasing, and the early-veg (September) events, where there was a marked decrease in repeatability for HoDs 13 and 14. The range in repeatability was greatest for the early-veg (September) events at HoD 9, ranging from 0.36 to 0.85.

### 3.6. Phenotypic Correlations for Airborne Canopy Temperature Within and Between Years

**Figure 7:**
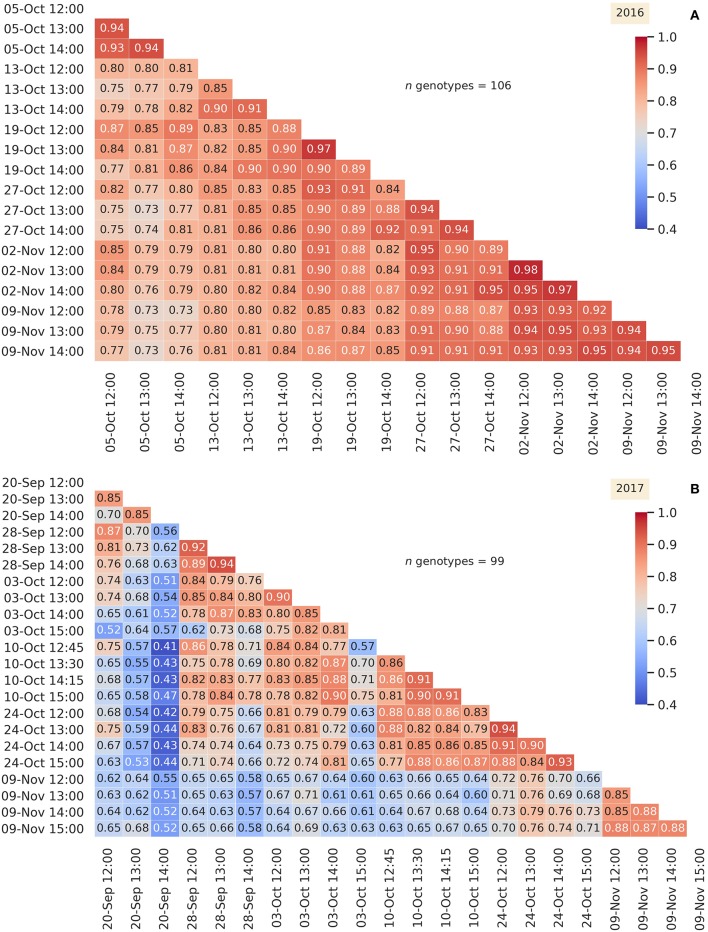
Heatmaps of phenotypic correlations between the best linear unbiased predictors of genotype effects (BLUPs) for selected airborne canopy temperature events in 2016 **(A)** and 2017 **(B)**. For 2016, on each day after (and including) 12:00. For 2017, for events on days after and including 20-Sept and after 12:00.

For both years, the phenotypic correlations between the best linear unbiased predictors of genotype effects (BLUPs) for airborne CT events were generally lower for HoDs before 12:00 and in 2017, for days before 20-September (pre-flowering). For these reasons, Figure 7 shows phenotypic correlations between BLUPs for selected airborne CT events in 2016 and 2017: for 2016, on each day after (and including) 12:00; for 2017, for events on days after and including 20-September and after 12:00. Frequency distributions of the selected airborne CT events are shown in Figure S18a for 2016 and Figure S18b for 2017. For the selected airborne CT events, the phenotypic correlations ranged from 0.73 to 0.98 in 2016, and from 0.41 to 0.94 in 2017. Phenotypic correlations between BLUPs for all airborne CT events are shown as frequency distributions (Figure S17a for 2016 and Figure S17c for 2017) and heatmaps (Figure S17b for 2016 and Figure S17d for 2017).

**Figure 8:**
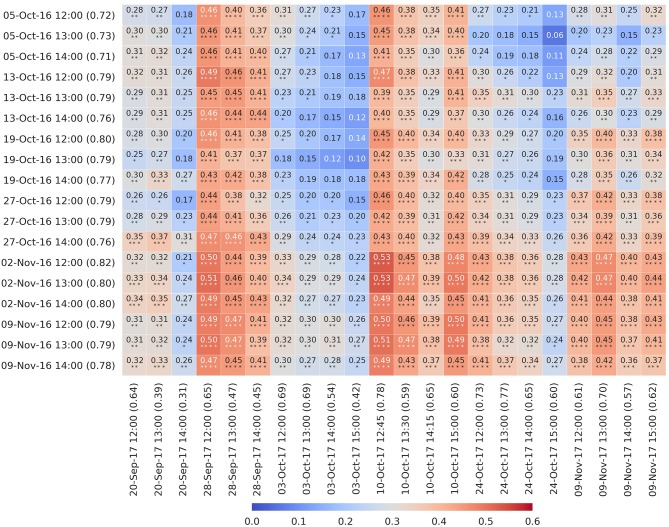
Heatmap of phenotypic correlations between the best linear unbiased predictors of genotype effects (BLUPs) (98 genotypes) for selected airborne canopy temperature (CT) events in 2016 and 2017 (as shown in Figure 7). For 2016, on each day after (and including) 12:00. For 2017, for events on days after and including 20-Sept-17 and after 12:00. Repeatability is shown in parenthesis for each respective airborne CT event. The two days in 2017 where the correlations were greatest (28-Sept-17 and 10-Oct-17), occurred soon after irrigation or rain (24 mm irrigation on 22-Sept-17 and 8 mm rain on 9-Oct-17) when the crop was less water-limited.

Phenotypic correlations across the years for BLUPs (for the 98 genotypes common to both years), for the 2016 and 2017 airborne CT events shown in Figure 7, are shown in Figure 8 (and frequency distribution in Figure S19). Figure 8 shows that phenotypic correlations between the selected 2016 and 2017 airborne CT events were moderate, ranging from 0.06 to 0.53, and greater on particular days in 2017 than others (e.g., 28-Sept-17, 10-Oct-17). For many of the individual CT events on 28-Sept-17 and 10-Oct-17, the phenotypic correlation was >0.40 and significantly associated (*P* < 0.0001) with every CT event in 2016, evidence of a strong genotypic effect across years. The two days in 2017 where the correlations were greatest (28-Sept-17 and 10-Oct-17), occurred soon after irrigation or rain (24 mm irrigation on 22-Sept-17 and 8 mm rain on 9-Oct-17) when the crop was less water-limited.

### 3.7. Association Between Repeatability and Weather Data

The scatter plot associations between CT repeatability estimates and the corresponding hourly weather data are shown in Figures S20–S23 for 2016 ArduCrop, 2016 airborne, 2017 ArduCrop and 2017 airborne, respectively. For 2016 ArduCrop CT, all weather variables were positively and significantly (*P* < 0.0001) associated with repeatability, with the calculated clear-sky solar radiation (*R*_*so*_), vapor pressure deficit (VPD) and the measured solar radiation (*R*_*s*_) at Griffith NSW (ca. 60 km north-west from the experiment site) having the strongest correlations with repeatability (0.61, 0.51, and 0.47, respectively) (Figure S20). For 2016 airborne CT all weather variables, except the wind parameters (average wind speed, maximum wind speed and wind direction), were positively and significantly (*P* < 0.01) associated with repeatability, with *R*_*so*_, *R*_*s*_ and VPD having the strongest correlations with repeatability (0.78, 0.45, and 0.44, respectively) (Figure S21). For 2017 ArduCrop CT, *R*_*so*_, *R*_*s*_, air temperature and VPD were positively and significantly (*P* < 0.0001) associated with repeatability (respective correlations with repeatability were 0.30, 0.27, 0.25, 0.21) (Figure S22). For 2017 airborne CT, only air temperature and VPD were significantly (*P* < 0.05) associated with repeatability with correlations of 0.39 and 0.39, respectively (Figure S23). Non-significant correlations between repeatability and *R*_*s*_, *R*_*so*_ were 0.30 and 0.27, respectively. Generally, correlations between CT repeatability and weather variables were greatest for *R*_*so*_, *R*_*s*_, VPD and air temperature. The association between CT repeatability and the wind parameters (average wind speed, maximum wind speed and wind direction) were generally poor and not significant, with the exception of 2016 ArduCrop where correlations were highly significant (*P* < 0.0001) and ranged from 0.24 to 0.28.

The day-of-year (DoY) and hour-of-day (HoD) were positively and significantly correlated with CT repeatability for 2016 ArduCrop CT (DoY: 0.47, *P* < 0.0001; HoD: 0.36, *P* < 0.0001), 2016 airborne CT (DoY: 0.38, *P* < 0.05; HoD: 0.44, *P* < 0.01) and 2017 ArduCrop CT (DoY: 0.14, *P* < 0.01; HoD: 0.18, *P* < 0.0001). For 2017 airborne CT, DoY was significantly associated with repeatability (0.33, *P* < 0.05), however the association with HoD was poor (0.02) and not significant.

Ordinary least squares (OLS) model results for estimates of CT repeatability (response variable) and the power set of the most significantly correlated weather (explanatory) variables (namely *R*_*so*_ and VPD) are shown in Table 2. Air temperature and *R*_*s*_ were omitted from the OLS models due to their high correlation with VPD and *R*_*so*_, respectively. Both DoY and HoD are directly related to *R*_*so*_ and were therefore modeled separately (discussed below). The wind parameters (average wind speed, maximum wind speed and wind direction) were also omitted from the models due to their generally poor correlation with repeatability (Figures S20–S23). Table 2 shows that, when modeled alone *R*_*so*_ and VPD were highly significant (*P* < 0.0001) and that the coefficient of determination (*R*^2^) was higher for *R*_*so*_ than VPD for ArduCrop and airborne in both years. Although VPD was highly significant (*P* < 0.0001) when modeled alone, the addition of VPD to *R*_*so*_ did not substantively increase the *R*^2^. Specifically, the addition of VPD to *R*_*so*_ increased the *R*^2^ from 0.815, with *R*_*so*_ alone, to 0.830 and from 0.990 to 0.992 for 2016 ArduCrop and airborne CT, respectively. For 2017 ArduCrop and airborne CT, the addition of VPD to *R*_*so*_ was non-significant. The OLS model results were consistent for ArduCrop and airborne in both years, where the ranking of models by *R*^2^ and the ranking of P-values for each respective model and variable were the same. In summary, the *R*^2^ values were high, ranging from 0.666 (2017 ArduCrop, VPD) to 0.992 (2016 airborne, *R*_*so*_ and VPD).

**Table 2:** Ordinary least squares model results for canopy temperature (CT) repeatability (response variable) and the weather (explanatory) variables most significantly and strongly correlated with repeatability, namely *R*_*so*_ and VPD (positive associations always).

		**Variables**		***P*-values**		***R*^2^**
				**VPD**	***R*_*so*_**	
		*R*_*so*_			^****^	0.815
2016 ArduCrop CT		VPD		^****^		0.707
		VPD	*R*_*so*_	^****^	^****^	0.830
		*R*_*so*_			^****^	0.990
2016 Airborne CT		VPD		^****^		0.851
		VPD	*R*_*so*_	^*^	^****^	0.992
		*R*_*so*_			^****^	0.792
2017 Airborne CT		VPD		^****^		0.666
		VPD	*R*_*so*_	ns	^****^	0.793
		*R*_*so*_			^****^	0.943
2017 Airborne CT		VPD		^****^		0.874
		VPD	*R*_*so*_	ns	^****^	0.943

Air temperature and R_s_ were omitted from the models due to their high correlation with VPD and R_so_, respectively. Statistically significant P-values are denoted:

**** P < 0.0001;

***P < 0.001;

**P < 0.01;

* *P < 0.05; ns, not significant. Scatter plot associations between CT repeatability estimates and corresponding hourly weather data are shown in Figures S20–S23 for 2016 ArduCrop, 2016 airborne, 2017 ArduCrop and 2017 airborne, respectively*.

Given that *R*_*so*_ is directly related to DoY and HoD, the latter two were modeled with VPD to test for their significance as surrogates for physiological effects (Table S2). For 2016 ArduCrop, the addition of VPD to DoY and HoD increased the *R*^2^ from 0.763 to 0.804. However, for 2016 and 2017 airborne, the addition of VPD to DoY and HoD did not increase the *R*^2^. Similarly for 2017 ArduCrop, the *R*^2^ only increased marginally from 0.792 (DoY and HoD) to 0.793 (VPD, DoY and HoD).

### 3.8. Association Between ArduCrop Canopy Temperature and Airborne Canopy Temperature

**Figure 9:**
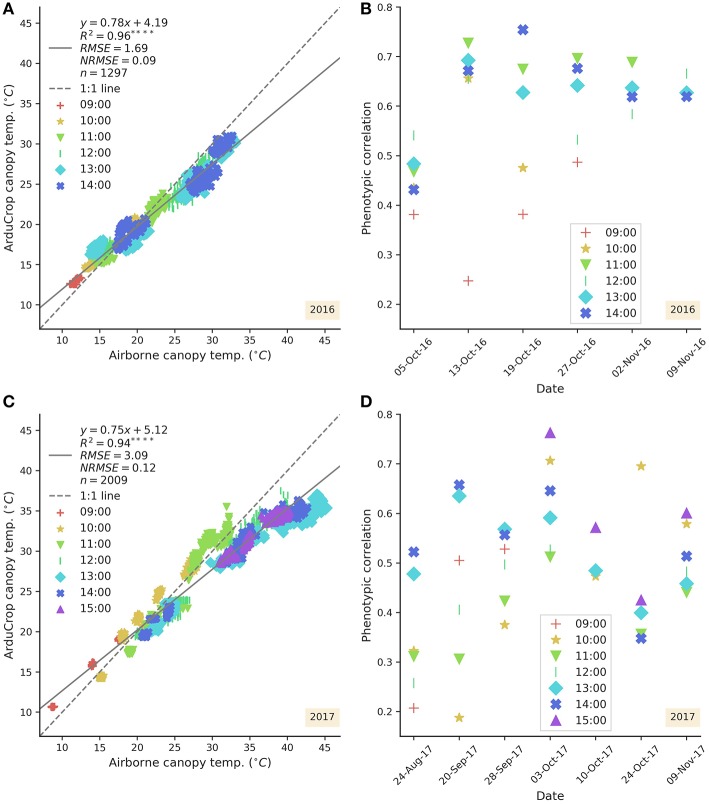
Association across all dates between airborne canopy temperature (CT) and ArduCrop CT (where for a given airborne CT event, data was compared to the nearest hourly ArduCrop CT event). Scatter plots for 2016 **(A)** and 2017 **(C)**. Scatter plots of phenotypic correlations between airborne CT and ArduCrop CT for each date 2016 **(B)** and 2017 **(D)**. Data are coded by hour-of-day as per respective legend. CT data are best linear unbiased predictors of genotype effects (BLUPs).

Airborne CT occurred on six and eight separate days in 2016 and 2017, respectively. In 2016 airborne CT commenced post-flowering in early October and in 2017, commenced pre-flowering in late August. Regressing the best linear unbiased predictors of genotype effects (BLUPs) for airborne and ArduCrop CT from all of the date-by-time events revealed a strong and significant association between the airborne and ArduCrop CT in 2016 (*R*^2^ = 0.96, *P* < 0.0001) and 2017 (*R*^2^ = 0.94, *P* < 0.0001) (Figures 9A,C for 2016 and 2017, respectively). In 2016, for temperatures >18°*C*, the ArduCrop CT was typically cooler than the airborne CT (slope of 0.78 and intercept of 4.19°*C*) while in 2017, for temperatures >21°*C*, the ArduCrop CT was typically cooler than the airborne CT (slope of 0.75 and intercept of 5.12°*C*). Figures 9B,D show that phenotypic correlations between airborne and ArduCrop CT BLUPs were typically higher in 2016 than 2017. In 2016, the phenotypic correlations between airborne and ArduCrop CT BLUPs ranged from 0.25 to 0.75 and with the exception of one event (13-Oct-16 at 09:00), ranged from 0.38 to 0.75. In 2017, the phenotypic correlations between airborne and ArduCrop CT BLUPs ranged from 0.19 to 0.76.

On an individual plot basis, the frequency distributions of phenotypic correlations between airborne and ArduCrop CT, show that the associations were generally greater in 2016 than 2017 (Figures S5a,b). In 2016, associations were highest from 11:00 onwards (with the exception of one event at 11:00) (Figure S5c). In 2017, the correlations were highest at 14:00 and 15:00 and, with the exception of two events at 10:00, tended to increase with hour-of-day (HoD) (Figure S5d). The same data are shown as scatter plots for each airborne CT event in Figures S3, S4, for 2016 and 2017, respectively.

## 4. Discussion

There is substantive evidence for the concomitant yield improvement in C3 crops and increased stomatal conductance in irrigated or non water-limited environments (for many examples see Roche, 2015). In turn, CT provides a surrogate measure of stomatal aperture traits, particularly stomatal conductance (Blum et al., 1982; Smith et al., 1988; Amani et al., 1996; Fischer et al., 1998; Jones, 2004; Jones and Vaughan, 2010; Rebetzke et al., 2013b). In this study, the consistently high estimates of CT repeatability obtained during grain-filling and from the middle of the day onwards (Figures 3, 6), together with the high phenotypic correlations between different sampling events (Figures 4, 5, 7, 8), provide confidence in the repeatability of CT phenotyping. These findings, together with the recent developments in reliable CT phenotyping through airborne thermography (Deery et al., 2016), provide further support for the use of CT to reliably screen germplasm, in both research and plant breeding, from as little as one or two sampling events.

### 4.1. Extreme Contrast in Available Soil Water Between 2016 and 2017

There was an extreme contrast in available soil water between 2016 and 2017, whereby the total rainfall and irrigation in 2017 was 283 mm less than that in 2016. The VPD was also greater in 2017 than 2016 (Table 1). The impacts of the contrast in available soil water between years are evident in nearly all of the results presented herein. Canopy temperature (CT) delta from air temperature (CT minus air temperature) plotted as a function of VPD has been used to indicate the crop water stress (e.g., Jackson et al., 1981; Idso et al., 1984), with higher values of CT delta denoting greater water stress. Table 1 shows that, for both ArduCrop and airborne CT, CT deltas during the grain-filling period were greater for 2017 than 2016, presumably due to reduced stomatal conductance and transpiration arising from the greater water limitation in 2017.

The assessments of CT delta enable comparison with those reported by Smith et al. (1988) where CT was measured continuously using a logging infra-red thermometer on wheat, well-watered and under drought, at Griffith NSW (*ca*. 60 km from the present study). Smith et al. (1988) reported a diurnal time-course of CT delta and VPD on 31-Oct-1985 and the approximate values at 13:00 h were as follows: CT delta of 2.0°C for well-watered; CT delta of 5.0 to 6.0°C under drought; VPD of 1300 *Pa*. The ArduCrop CT delta under well-watered conditions in 2016 was 1.0°C (Table 1), consistent with those reported by Smith et al. (1988) for well-watered wheat during grain-filling. By contrast, the ArduCrop CT delta under water limited conditions in 2017 was 6.8°C, slightly greater than the range of 5.0 to 6.0°C reported by Smith et al. (1988) for their drought treatment. Together these findings provide confidence in the ArduCrop method. By contrast to the VPD and CT deltas from air temperature reported herein (and in Smith et al., 1988), the VPD reported from CT studies in Mexico and Arizona were much greater and CT was typically less than air temperature by up to 5°C or more in well-watered experiments (Jackson et al., 1981; Idso et al., 1984; Amani et al., 1996).

### 4.2. Comparison of ArduCrop and Airborne Canopy Temperature

That the variance for the internal ArduCrop replication in 2016 was negligibly small (Figure S1), indicates that the additional ArduCrop sensors on the duplicate-ArduCrop-plots did not significantly improve the estimate of CT. However, the highly significant association between the internal ArduCrop replicate sensors (*R*^2^ = 0.98, *P* < 0.0001), together with the slope of 0.98 and intercept of 0.34 (Figure 2), provides good evidence for the capacity of an individual ArduCrop sensor to reliably measure CT.

For CT >*ca*. 20°*C*, the ArduCrop CT was typically cooler than the airborne CT across both years (Figures 9A,C for 2016 and 2017, respectively). This can be partly attributed to the respective viewing angles of the ArduCrop and airborne CT methods. The ArduCrop viewing angle was 45° (facing downward) to minimize the likely influence of soil temperature resulting from the 25 cm row spacing used herein. In contrast, the airborne CT viewing angle was nadir (directly above) and therefore likely to sample a greater proportion of soil than the ArduCrop. For a given plot, the airborne CT was derived from the mean of all pixels within a rectangle and no attempt was made to remove temperature pixels resulting from the background soil. The latter is because a previous study at the Yanco site, using the same row spacing of 25 cm, found that methods based on filtering the frequency distribution of the temperature pixels to remove the influence of background soil did not improve the estimates of broad-sense heritability (Deery et al., 2016). Nevertheless, the airborne CT as measured from the nadir view, was possibly influenced by the degree of fractional cover and the soil temperature. In our study, early morning airborne CT measurements at HoD 9 were typically cooler than ArduCrop CT measurements and conversely, airborne CT measurements from midday onwards were often warmer than ArduCrop CT (Figure 9). These differences may have been attributable to the airborne CT sampling a cooler soil temperature in the morning and a warmer soil temperature from midday onwards. For a given event the association between ArduCrop and airborne CT BLUPs (Figures 9B,D) tended to improve with HoD, for both years, and was generally greater in 2016 than 2017. That for a given experimental plot the sampling area of the ArduCrop (*ca*. 0.02 mʦ2) was far less than for airborne CT (*ca*. 4.0 mʦ2), the reasonable association between ArduCrop and airborne CT BLUPs is encouraging and provides confidence in the precision of both methods.

### 4.3. Higher Repeatability for Canopy Temperature During Grain-Filling and From 11:00 Onwards

The repeatability of CT was typically greater during the grain-filling growth stage in October and November than during the pre-flowering months of August and September (Figures 3, 6). The ArduCrop CT repeatability estimates were often greater from 11:00 onwards in 2016 (Figure 3A) and 2017, although less so in 2017 (Figure 3B). These patterns were more pronounced in the more favorable growing environment of 2016 than the more water-limited environment of 2017 (cf. 2016 ArduCrop data (Figure 3A) with the 2017 ArduCrop data (Figure 3B). The repeatability estimates for airborne CT were slightly lower for events at 09:00 during 2016 (Figure 6A), however no clear HoD pattern was evident in 2017 (Figure 6B). The greater repeatability of CT later in the season is consistent with previous studies including Rutkoski et al. (2016), where in four out of their five environments, broad-sense heritabilities of CT on a line mean basis and on a single plot basis, were greater during grain-filling than pre-flowering. The higher repeatability for ArduCrop CT from 11:00 onwards, particularly in 2016, is similar to experiments with Pima cotton, whereby the optimal time for screening stomatal conductance was two to three hours past solar noon (Lu et al., 1998). Similarly in a selection of 23 spring wheat cultivars (Amani et al., 1996), correlations between CT and yield were greatest for CT measurements made between noon and 4pm compared to those made between 8am and noon. Further, in a recent study involving continuous CT measurements on 20 winter wheat cultivars (Thapa et al., 2018), variation between genotypes was greater during the middle of the day than early morning.

Ordinary least squares modeling revealed that the calculated clear-sky solar radiation (*R*_*so*_) and vapor pressure deficit (VPD) were highly significant explanatory variables for repeatability (Table 2), with *R*_*so*_ the most highly significant explanatory variable for ArduCrop and airborne CT in both years. The addition of VPD to the model comprising either *R*_*so*_ or its determinants, day-of-year and hour-of-day, made little to no improvement to the coefficient of determination (Table S2). Thus, it is possible that genotypic differences in CT, and potentially stomatal conductance (Rebetzke et al., 2013b), were more pronounced during the grain-filling stage because of greater solar radiation and VPD. From energy balance theory linking the estimation of CT from the local weather variables (e.g., Jackson et al., 1981; Smith et al., 1988; Jones and Vaughan, 2010), for a given stomatal conductance, CT is linearly related to solar radiation and VPD. Therefore, it seems biophysically plausible that genotypic differences in stomatal conductance would produce larger differences in CT, and potentially (but not necessarily) repeatability, with greater solar radiation and VPD, both of which are more likely to occur during grain-filling and later in the day. That CT repeatability was generally lower earlier in the day is not surprising given that solar radiation and VPD are lower earlier in the day. However, that the most significant explanatory variable associated CT repeatability was *R*_*so*_, a diurnal function that increases with the day-of year-after the winter solstice in the southern hemisphere, implies a significant temporal association with CT repeatability that may be mechanistic in nature. Although the mechanisms responsible for the apparent increase in CT repeatability during grain-filling cannot be identified with certainty, possible reasons include: (a) that genotypes differed in their capacity to extract water from the soil with cooler genotypes producing deeper root systems (e.g., Lopes and Reynolds, 2010; Pask and Reynolds, 2013); (b) that genotypes with cooler canopies were responding to either higher photosynthetic capacity or higher sink demand for photosynthate - for example Tang et al. (2015, 2017) reported that genotypes with greater canopy photosynthesis, measured at flowering and 20 days after flowering, had greater leaf chlorophyll (as measured by SPAD) and were also higher yielding; (c) that the genotypes differed in “stay-green” (a genotype's capacity to continue assimilating carbon toward the latter part of grain-filling) and that such differences increased as they moved into grain-filling, so that cooler genotypes had greater green leaf area (e.g., Christopher et al., 2016; Rebetzke et al., 2016); and (d) that the genotypes differed in their seasonal pattern of water-use, so that cooler genotypes had lower water-use pre-flowering and greater water-use post-flowering (e.g., Richards and Passioura, 1989; Rebetzke et al., 2003; Blum, 2005).

Although in our experiments the repeatability estimates for CT were often smaller pre-flowering, the importance of stomatal conductance during the pre-flowering growth stage was recently highlighted by Motzo et al. (2013), whereby the greater pre-flowering radiation-use-efficiency of triticale was associated with greater stomatal conductance and greater biomass than durum wheat. Although pre-flowering genotypic variation for stomatal conductance may be potentially useful for yield improvement, our experiments suggest that detecting such variation using CT as a surrogate measure of stomatal conductance may be difficult. This is because of the likely smaller differences in conductance pre-flowering, due to lower VPD and solar radiation, and therefore lower sensitivity of CT to conductance. The latter would likely result in reduced repeatability of CT. Published theoretical calculations and sensitivity analyses are useful for understanding the relationship between CT and conductance for a range of weather variables (Leinonen et al., 2006; Maes and Steppe, 2012). In particular, theoretical calculations for a range of conductance values show the convergence of CT with decreasing VPD (Maes and Steppe, 2012, their Figure 3), thereby highlighting the potential difficulty in detecting variation in CT at low VPD. Error analysis showing the steep increase in relative error with decreasing conductance (Leinonen et al., 2006, their Figure 4), further highlights the challenge with detecting variation for conductance using CT at low conductance values. However, that our results show a strong phenotypic correlation between CT measurements, more so when repeatability was high (Figure S11), suggests the possibility of a sufficient phenotypic correlation between pre and post-flowering CT for screening purposes (discussed later).

### 4.4. High Phenotypic Correlation When Repeatability Was High

The phenotypic correlation between the best linear unbiased predictors of genotype effects (BLUPs), for both ArduCrop and airborne CT within a particular year, was high when repeatability and confidence in among-genotypic differences was high. For the ArduCrop CT, the mean, median and percentiles of phenotypic correlations all increased with repeatability quantiles (i.e., 0.0 to 0.33 < 0.33 to 0.66 < 0.66 to 1.0) in both years (Figure S11). In particular, for the 2016 ArduCrop repeatability quantile 0.66 to 1.0, the median phenotypic correlation was 0.84 and the 25ʦth percentile was 0.74 (Figure S11c).

The high phenotypic correlations for the airborne CT in 2016 (Figure 7A) provide evidence of the repeatability of CT measurements between different sampling events. Together these results provide confidence in the potential for CT phenotyping, whereby the sufficiently high repeatability and phenotypic correlation across multiple sampling events, for afternoon events later in the season, could permit reliable genotype screening from as little as one or two sampling events provided soil water availability is not constrained.

That the phenotypic correlations between the 2016 and 2017 airborne CT events were typically smaller (median was 0.32 shown in Figure S19) is not surprising given the contrast in available water between the two years. Although the correlations for airborne CT between years were moderate, ranging from 0.06 to 0.53, they were greater on particular days in 2017 than others, in particular 28-Sept-17 and 10-Oct-17 (Figure 8) when the crop was less water-limited (24 mm irrigation applied on 22-Sept-17 and 8 mm rain on 9-Oct-17). The correlations on these two days, for the midday events, were highly significant (*P* < 0.0001), ranging from *r* = 0.39 to 0.53. The higher correlations after irrigation and rainfall events on 22-Sept-17 and 9-Oct-17, between two environments with an extreme contrast in available water throughout the season, provide confidence in the capacity of CT to reliably discriminate genotypes in a generally water-limited environment (2017), provided that CT is sampled soon after an irrigation or rainfall event when the soil water stress is reduced. Nevertheless, that many of the individual CT events on 28-Sept-17 and 10-Oct-17 were significantly correlated with every CT event in 2016 (Figure 8), provides evidence of a strong genotypic effect across years and for the potential of CT in more favorable environments that are not exposed to severe water limitation (discussed below).

### 4.5. Implications for Research and Plant Breeding

The estimates of repeatability and phenotypic correlations for CT, across multiple sample events, were notably greater in the more favorable 2016 environment than those in the water-limited 2017 environment (Figures 3–7). Further, the phenotypic correlations across the years between selected 2016 and 2017 airborne CT events were greater on particular days in 2017 than others, probably due to the severe water limitation in 2017, while the 2016 events were devoid of such day effects (Figure 8). That the two days in 2017 when the correlations with 2016 events were greatest (*r* = 0.39 to 0.53, *P* < 0.0001) occurred soon after an irrigation or rainfall event, provides support for the use of CT as a selection tool in more favorable environments that are not exposed to severe water limitation. While such favorable environments may not represent the complete target population of environments for a breeding program, there is good evidence in wheat supporting the use of favorable selection environments (Cooper et al., 1997) and that yield potential progress can translate across to most environments, except those most strongly water-limited (Araus et al., 2002; Rebetzke et al., 2002; Olivares-Villegas et al., 2007).

The consistently high estimates of CT repeatability obtained herein and in previous studies (e.g., Deery et al., 2016; Rutkoski et al., 2016) is encouraging for the potential use of CT for indirect selection of grain yield in a breeder's nursery (Fischer and Rebetzke, 2018). This is because the theory of correlated response to indirect selection (Falconer, 1952) shows greater benefits for indirect selection when both the heritability for the indirect trait (i.e., CT) and the genetic correlation between the indirect and target trait (i.e., yield) are high. While a number of studies have reported high genetic correlations between CT and grain yield (e.g., Rebetzke et al., 2013b; Rutkoski et al., 2016), to the best of our knowledge few studies have reported such consistently high estimates of repeatability from multiple sampling events as those presented herein.

The interquartile range for CT variation between genotypes on a given sampling event was typically <1.0°C for both ArduCrop (Figures S9, S10) and airborne (Figures S13–S16). Thus, the high precision camera used herein, with <0.05°C pixel-to-pixel sensitivity, is ideally suited to the application of CT phenotyping. Further, by using a manned helicopter at an approximate height of 120 m above-ground-level, large image swaths were obtained: using the camera described above, at 120 m AGL, an image swath *ca*. 87.1 m by 64.1 m was obtained with a pixel size 0.14 × 0.13 m, which equated to *ca*. 55 temperature pixels per mʦ2. Such large swaths enabled sampling from the entire experiment, with dimensions of 50 × 110 m in 2016 and 25 × 110 m in 2017, in a few seconds, together limiting the impact of slight weather fluctuations to reduce experimental noise and increase the measurement precision of CT. While unmanned aerial vehicles (UAVs) have been used for thermal image acquisition (e.g., Sullivan et al., 2007; Berni et al., 2009a,b; Zarco-Tejada et al., 2012; Chapman et al., 2014; Gómez-Candón et al., 2016), their effectiveness for quantifying repeatable CT differences among genotypes is yet to be determined. To the best of our knowledge, no study has reported high estimates of CT repeatability or heritability from a UAV.

The ArduCrop sensors measure CT continuously on a single experimental plot at any given time. By contrast, the airborne method measures CT across large experiments comprising hundreds of plots at a single moment in time and, supported by the high repeatability estimates and phenotypic correlations herein, is ideally suited to deployment within plant breeding. Despite that in our experiments the repeatability estimates from ArduCrop CT were high during grain-filling and in the afternoon (Figure 3), the deployment of large numbers of ArduCrop sensors, in the numbers deployed herein (*ca*. 100), on a breeder's trial is not practically feasible nor justified when compared to the airborne method. However, the reasonable association between ArduCrop and airborne CT (Figure 9) is encouraging and provides confidence in the precision of the ArduCrop sampling only a fixed small part of each plot. This is further supported by the high correlation between the internal ArduCrop replicate sensors and the negligibly small variance for the internal ArduCrop replication in 2016 (Figure 2, Figures S1, S2 and Table S1). The use of the ArduCrop CT sensors is probably more suited to detailed crop physiology studies and applications where an understanding of the crop's diurnal and seasonal response to the environment is required. For example, the possibility to estimate crop canopy conductance and evaporation rate from ArduCrop CT (Jones et al., 2018) may assist with improved: environmental characterization using probe genotypes; agronomic decisions including irrigation scheduling (e.g., Mahan et al., 2012); characterization of frost or high temperature events.

Clearly in most cases, CT phenotyping is of diminishing value unless CT can be confidently related to stomatal conductance and yield. However, there is the possibility that variation in canopy structure (e.g., height, ground-cover, architecture and albedo) and stage of development (e.g., variation in flowering and maturity dates) can influence the association between stomatal conductance and CT. Moreover, sensitivity analyses indicate that variation in height, albedo, leaf area index and leaf angle can influence the relationship between CT and stomatal conductance (Maes and Steppe, 2012). Nevertheless, the effectiveness of UAV platforms for quantifying leaf area and plant height (Potgieter et al., 2017; Hu et al., 2018), together with ground-based platforms (e.g., Deery et al., 2014; Jimenez-Berni et al., 2018), presents an opportunity for greater understanding between conductance, CT and yield, in the presence of variation in canopy structure, and highlights the need for further work in this area.

## 5. Conclusions

Repeatability estimates for ArduCrop and airborne CT in wheat were typically greater later in the season during grain-filling and in the afternoon. This was supported by the observation that the pattern of repeatability, for ArduCrop and more so for airborne CT, was significantly associated (*P* < 0.0001) with the calculated clear-sky solar radiation and to a lesser degree, vapor pressure deficit. The latter is because the addition of vapor pressure deficit to a model comprising either clear-sky solar radiation or its determinants, day-of-year and hour-of-day, made little to no improvement to the coefficient of determination. For airborne CT afternoon sampling times, the phenotypic correlations were consistently high across sampling times within a given year and, to a lesser extent, between years contrasting in soil water availability. The phenotypic correlations for ArduCrop CT were higher during the grain-filling months of October and November and for hours-of-day from 11 onwards. In contrast, the lowest correlations comprised events from hours-of-day 8 and 9 across all months. These findings build upon the recent developments in CT phenotyping as a surrogate measure of stomatal conductance and the abundant evidence of the association between wheat yield improvement and high stomatal conductance. Together these factors provide promising support for the reliable deployment of CT phenotyping within both pre and commercial plant breeding, whereby the high repeatability and phenotypic correlation across afternoon sampling events later in the season could enable reliable screening of germplasm from as few as one or two sampling events.

## Author Contributions

GR selected the germplasm and designed the field experiments. DD analyzed the data and made the figures with input and advice from GR, RF, and SC. DD, GR, JJ-B, RJ, AC, WB, RF, and SC contributed to the conception of the study and the manuscript. DD wrote the manuscript with input from RF, GR, WB, JJ-B, RJ, and RF.

### Conflict of Interest Statement

The authors declare that the research was conducted in the absence of any commercial or financial relationships that could be construed as a potential conflict of interest.
