# Supplementary material for: Evaluation of the Phenotypic Repeatability of Canopy Temperature in Wheat Using Continuous-Terrestrial and Airborne Measurements
Source: Front Plant Sci. 2019 Jul 9;10:875. doi: 10.3389/fpls.2019.00875 (PMC6629910; doi:10.3389/fpls.2019.00875)
Supplement: Supplementary file 1 [file Data_Sheet_1.pdf]

Supplementary Material:
Evaluation of the phenotypic repeatability of canopy temperature in wheat using continuous-terrestrial and airborne measurements

David M. Deery*, Greg J. Rebetzke, Jose A. Jimenez-Berni, William D. Bovill, Richard A. James, Anthony G. Condon, Robert T. Furbank, Scott C. Chapman, Ralph (Tony) A. Fischer

*Correspondence:
Author Name: David M. Deery
david.deery@csiro.au

1 FIGURES

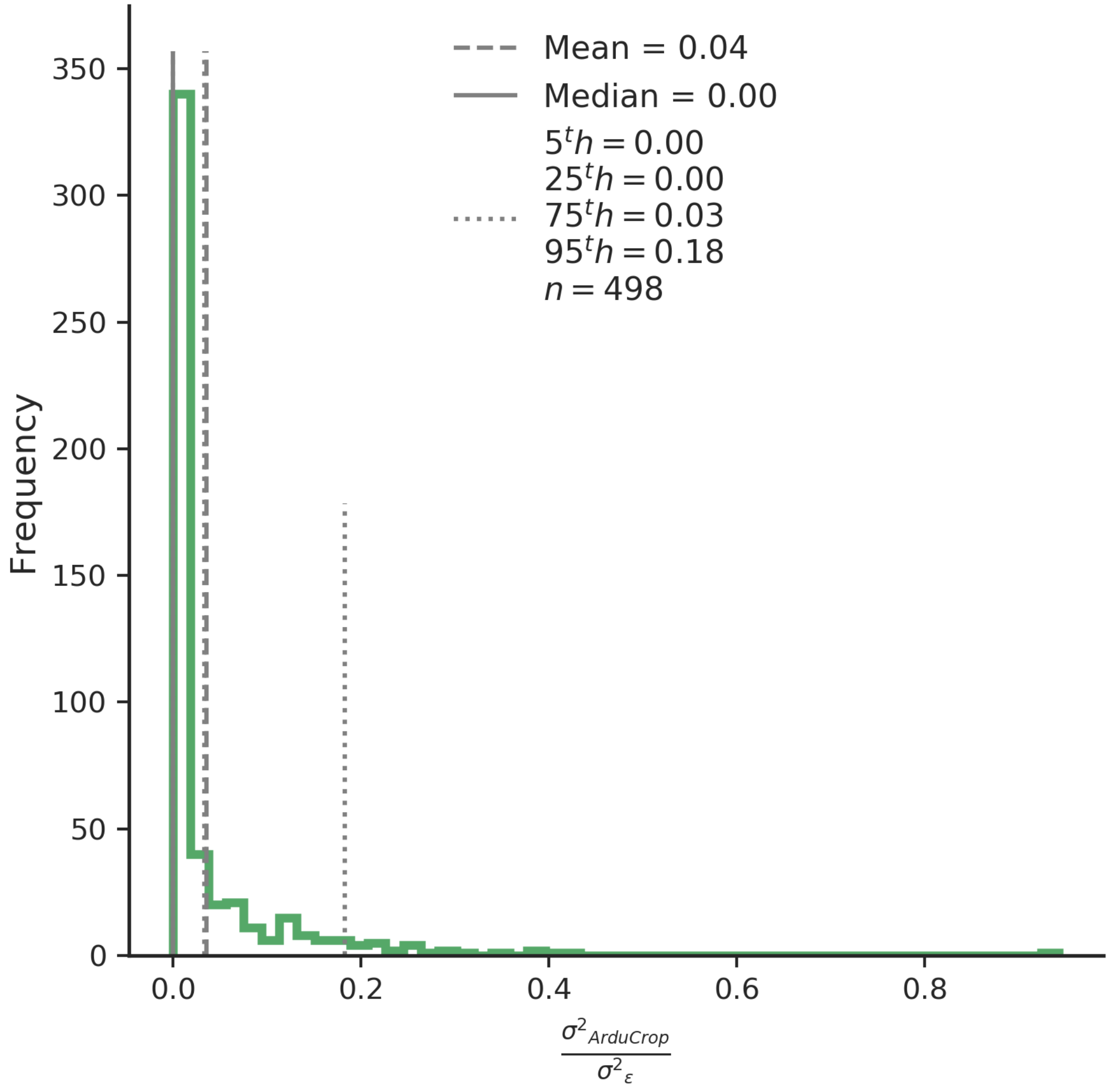

Figure S1. Frequency distribution of variance ratios for the internal ArduCrop replication: $\frac{\sigma^2{}_{ArduCrop}}{\sigma^2{}_{\epsilon}}$. Where $\sigma^2{}_{ArduCrop}$ and $\sigma^2{}_{\epsilon}$ are the internal ArduCrop replicate and residual variances, respectively. The percentile score denoting when the variance ratios, $\sigma^2{}_{ArduCrop}$ and $\sigma^2{}_{\epsilon}$, were less than 0.1 was 87.

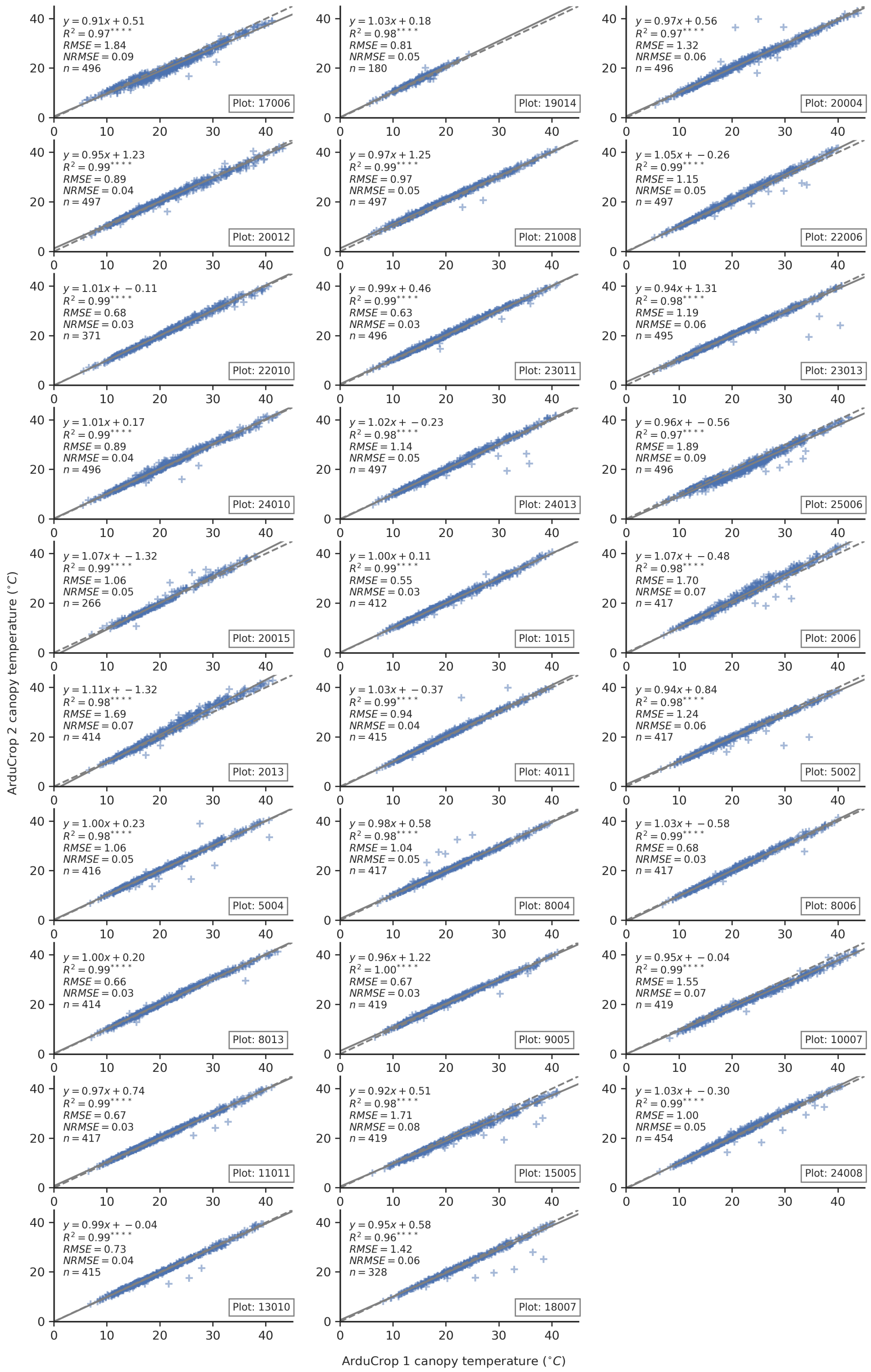

Figure S2. Scatter plot of ArduCrop 1 and ArduCrop 2 with fitted linear regression equation, coefficient of determination (R^2), root mean square error (RMSE), normalised RMSE (NRMSE) and the number of values (n) for each of the 29 plots that comprised two paired ArduCrop sensors in 2016 (i.e. internal ArduCrop replication denoted ArduCrop 1 and ArduCrop 2). Solid line denotes fitted linear regression and dashed line denotes 1:1 line.

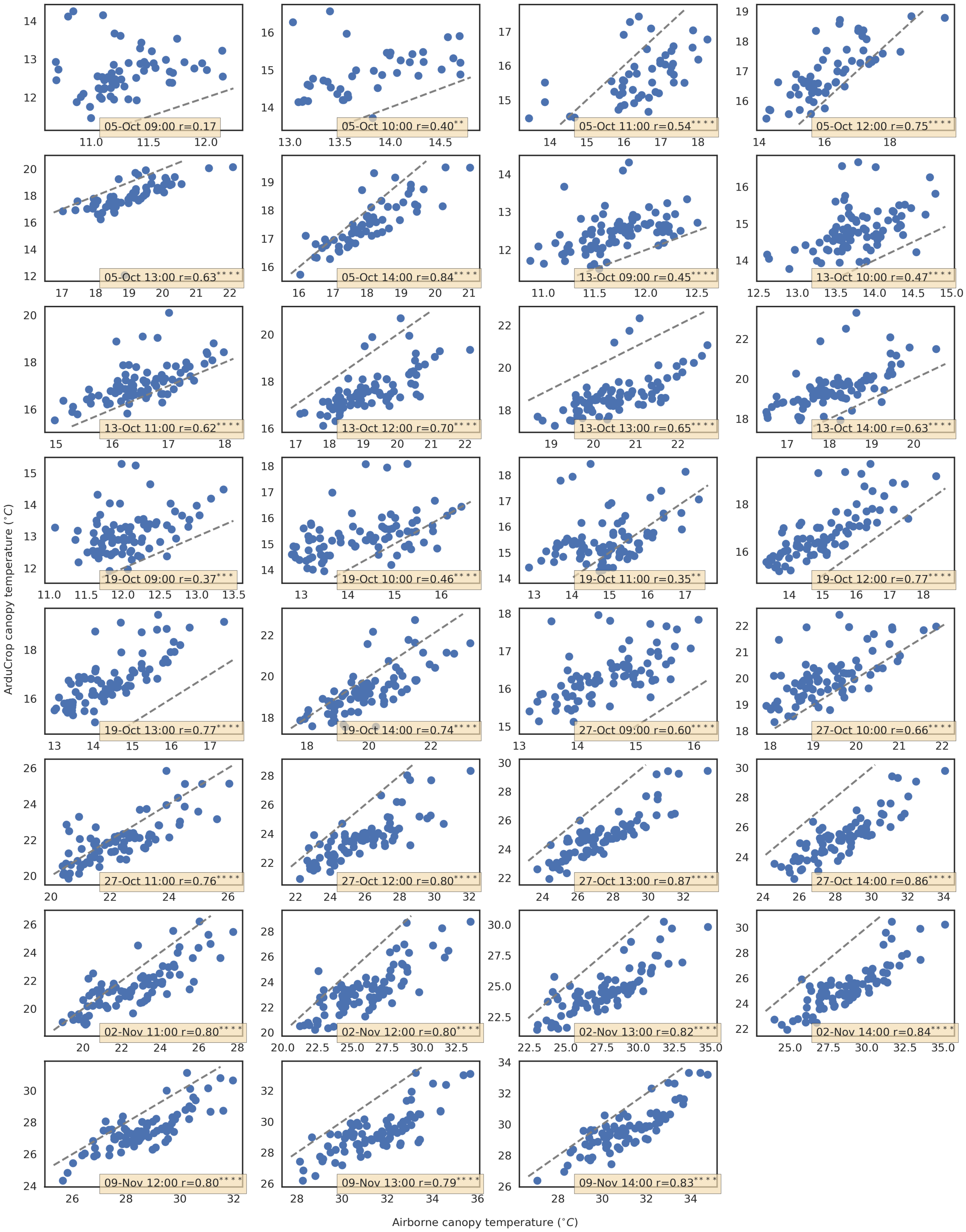

Figure S3. Association on an individual plot basis between airborne canopy temperature (CT) and ArduCrop CT in 2016. Pearson correlation, r, with statistically significant non-zero slopes denoted: ****, $P < 0.0001$; ***, $P < 0.001$; **, $P < 0.01$; *, $P < 0.05$.

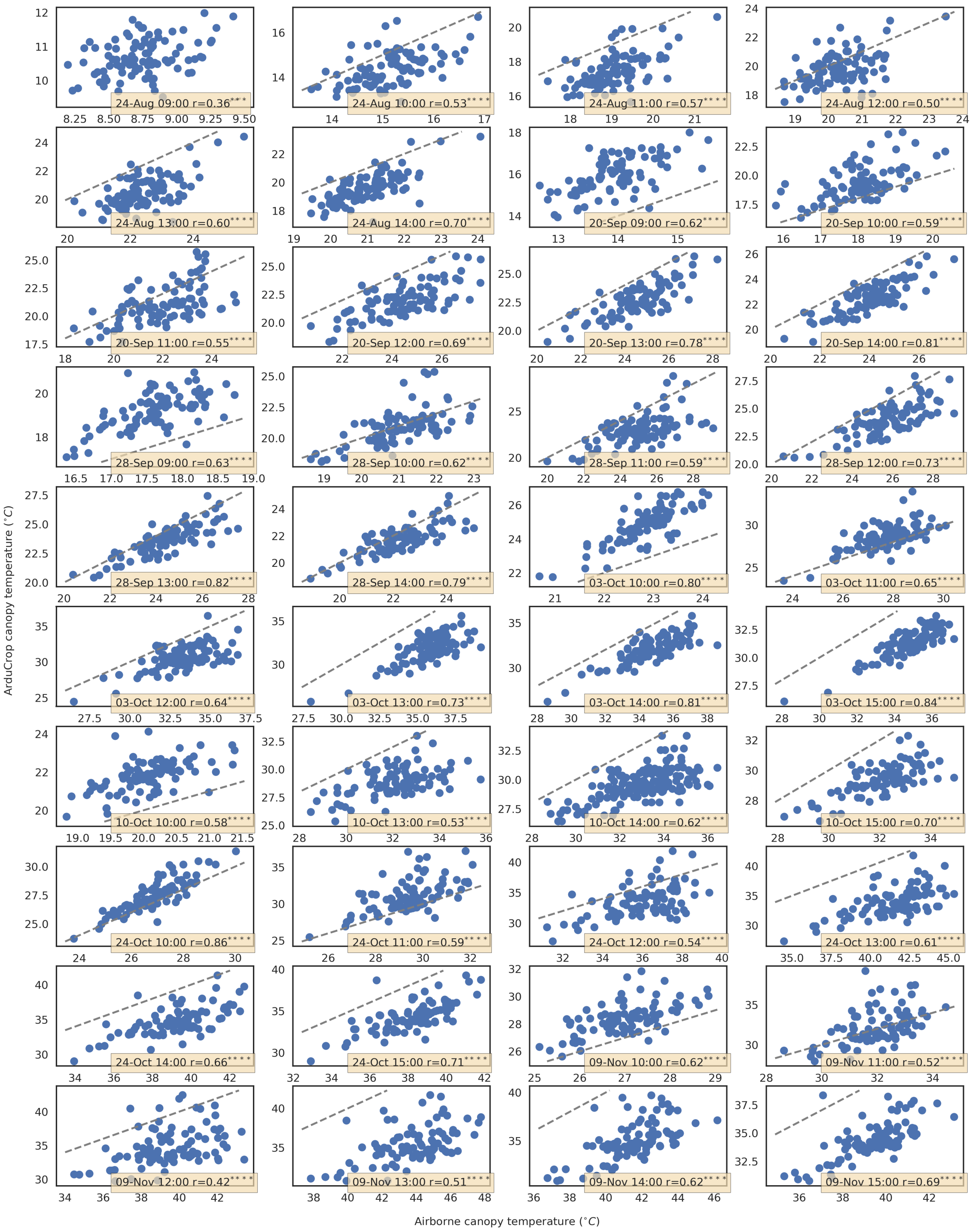

Figure S4. Association on an individual plot basis between airborne canopy temperature (CT) and ArduCrop CT in 2017. Pearson correlation, r, with statistically significant non-zero slopes denoted: ****, $P < 0.0001$; ***, $P < 0.001$; **, $P < 0.01$; *, $P < 0.05$.

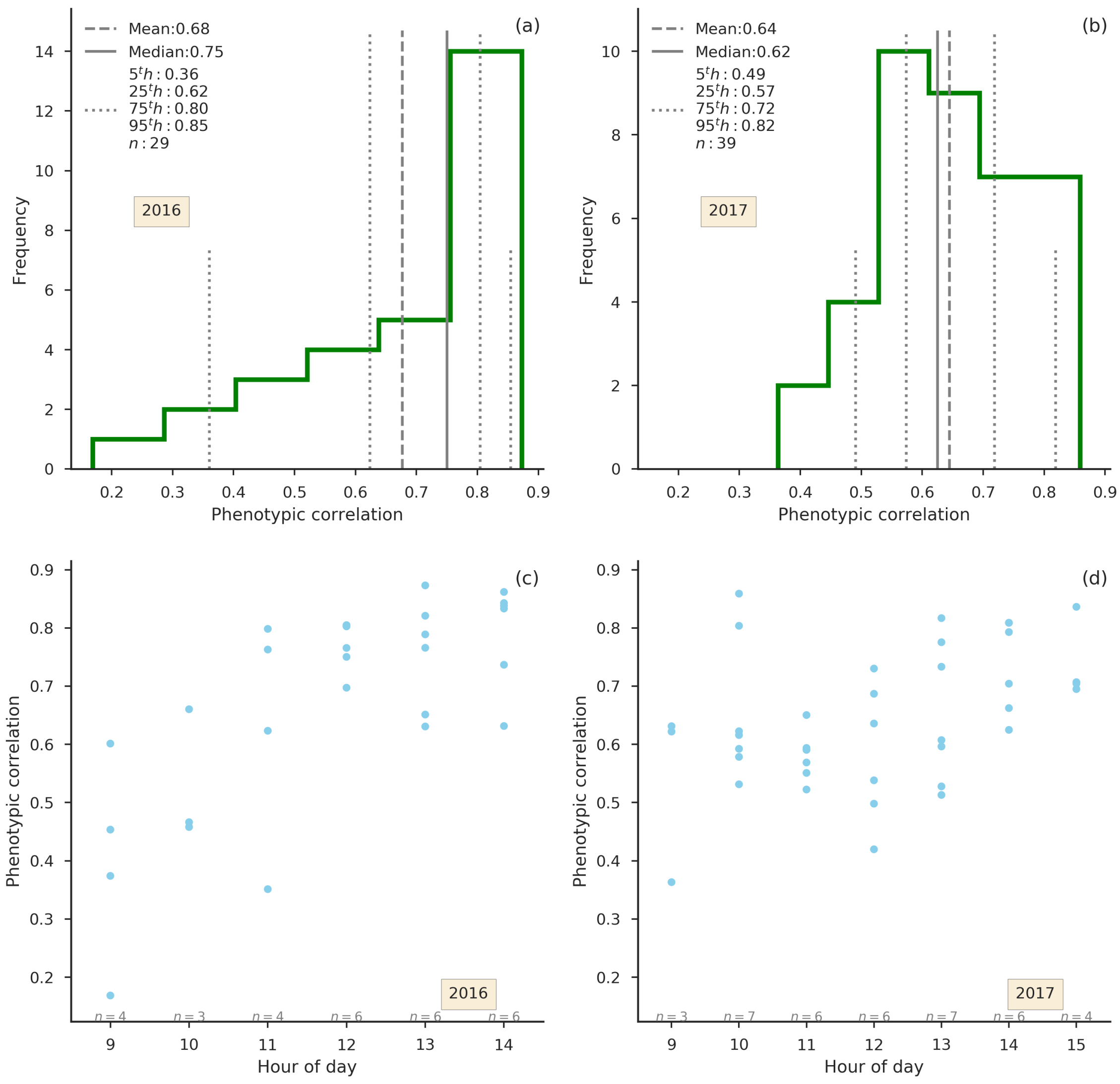

Figure S5. Summary of Pearson correlations between airborne canopy temperature (CT) and ArduCrop (CT) in 2016 and 2017 on an individual plot basis for the events shown in Figures S3 and S4. Frequency distribution for 2016 (a) and 2017 (b). Scatter plot grouped by hour of day for 2016 (c) and 2017 (d).

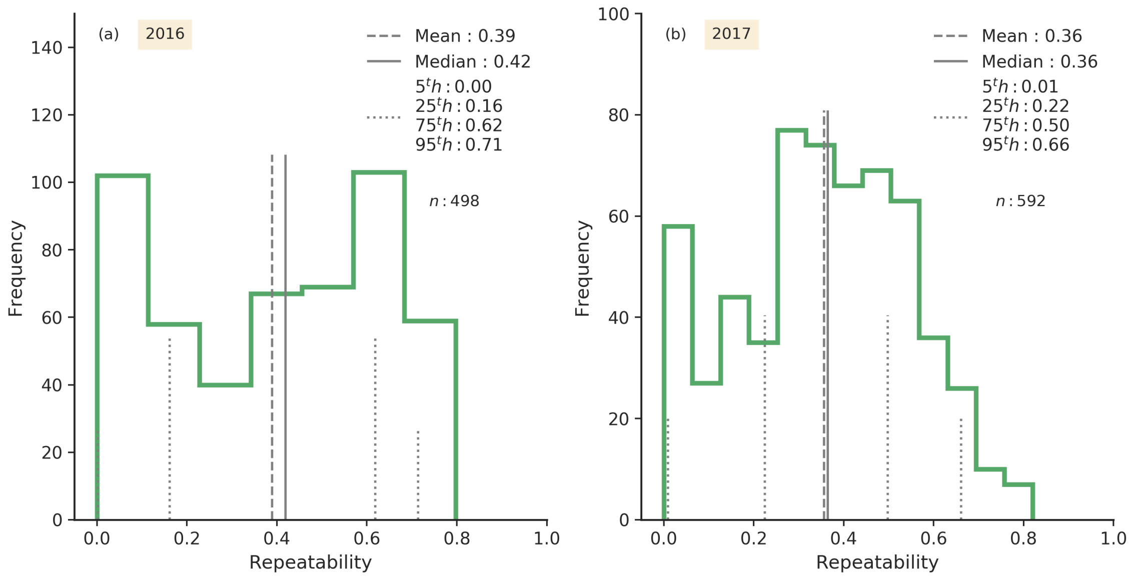

Figure S6. Frequency distributions of repeatability estimates for ArduCrop canopy temperature in 2016 (a) and 2017 (b).

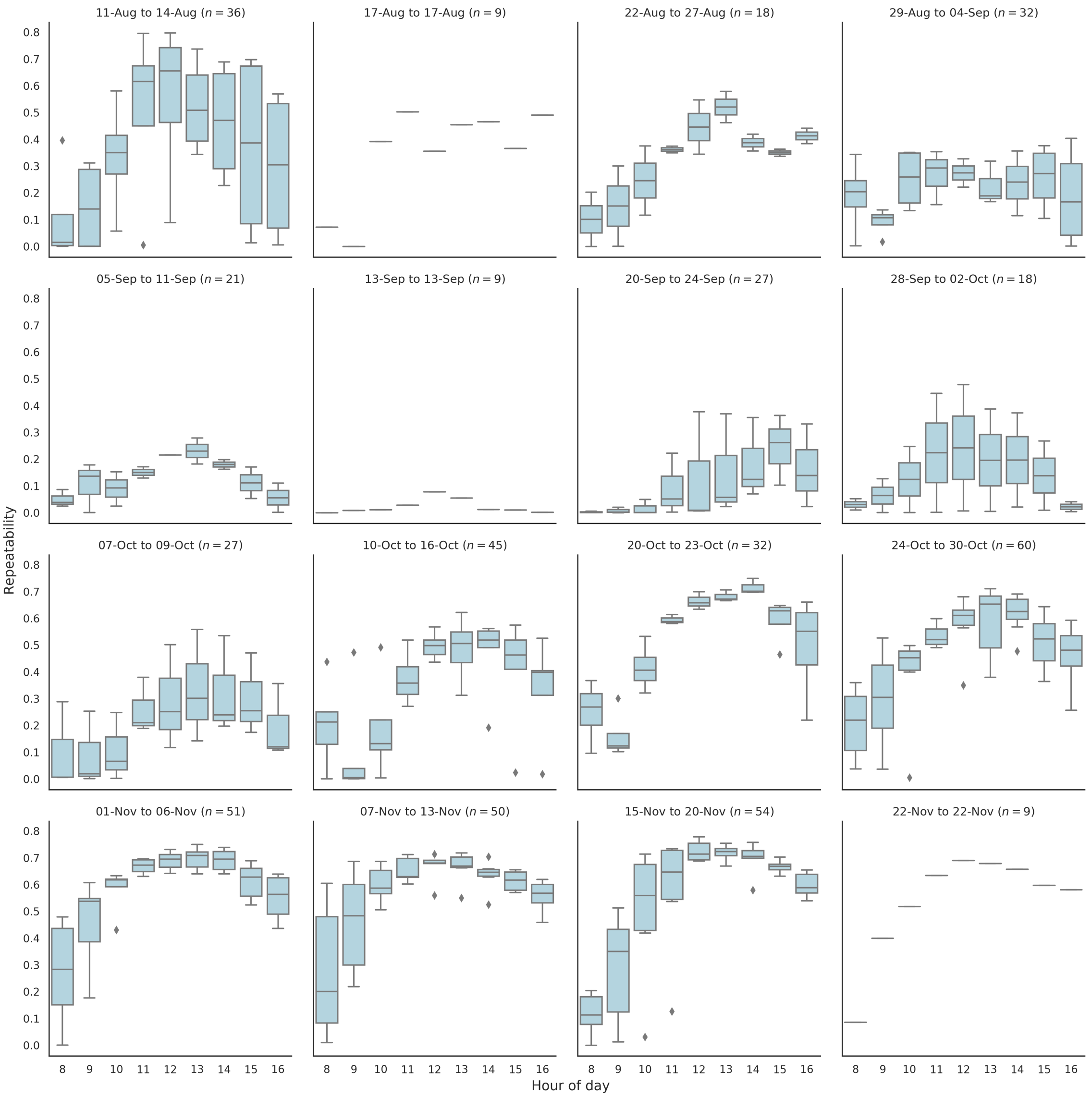

Figure S7. Summary of repeatability estimates for ArduCrop canopy temperature in 2016. Box plots of repeatability estimates for each hour of the day grouped by week. The number of repeatability estimates in each sub-figure is denoted n.

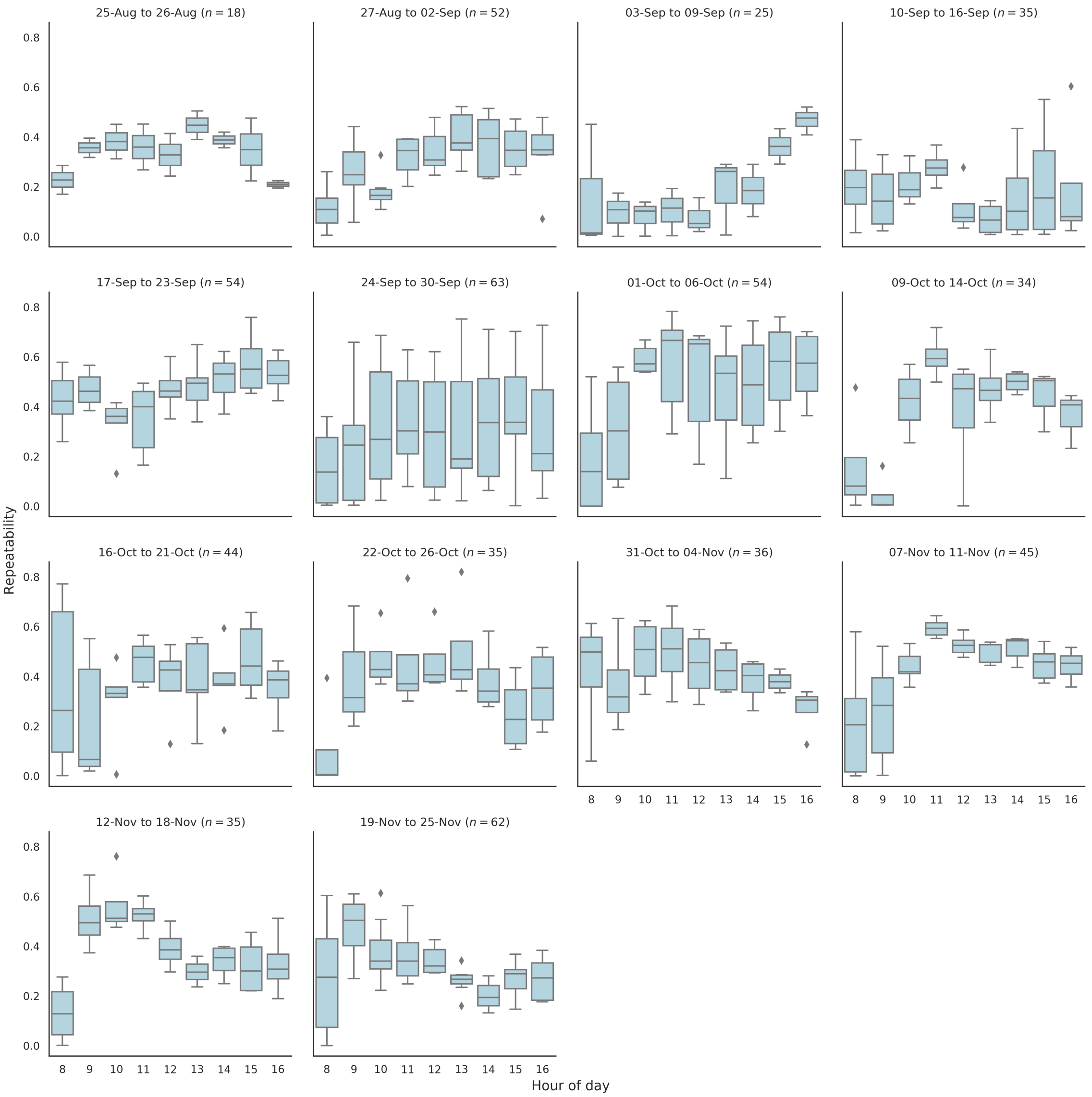

Figure S8. Summary of repeatability estimates for ArduCrop canopy temperature in 2017. Box plots of repeatability estimates for each hour of the day grouped by week. The number of repeatability estimates in each sub-figure is denoted n.

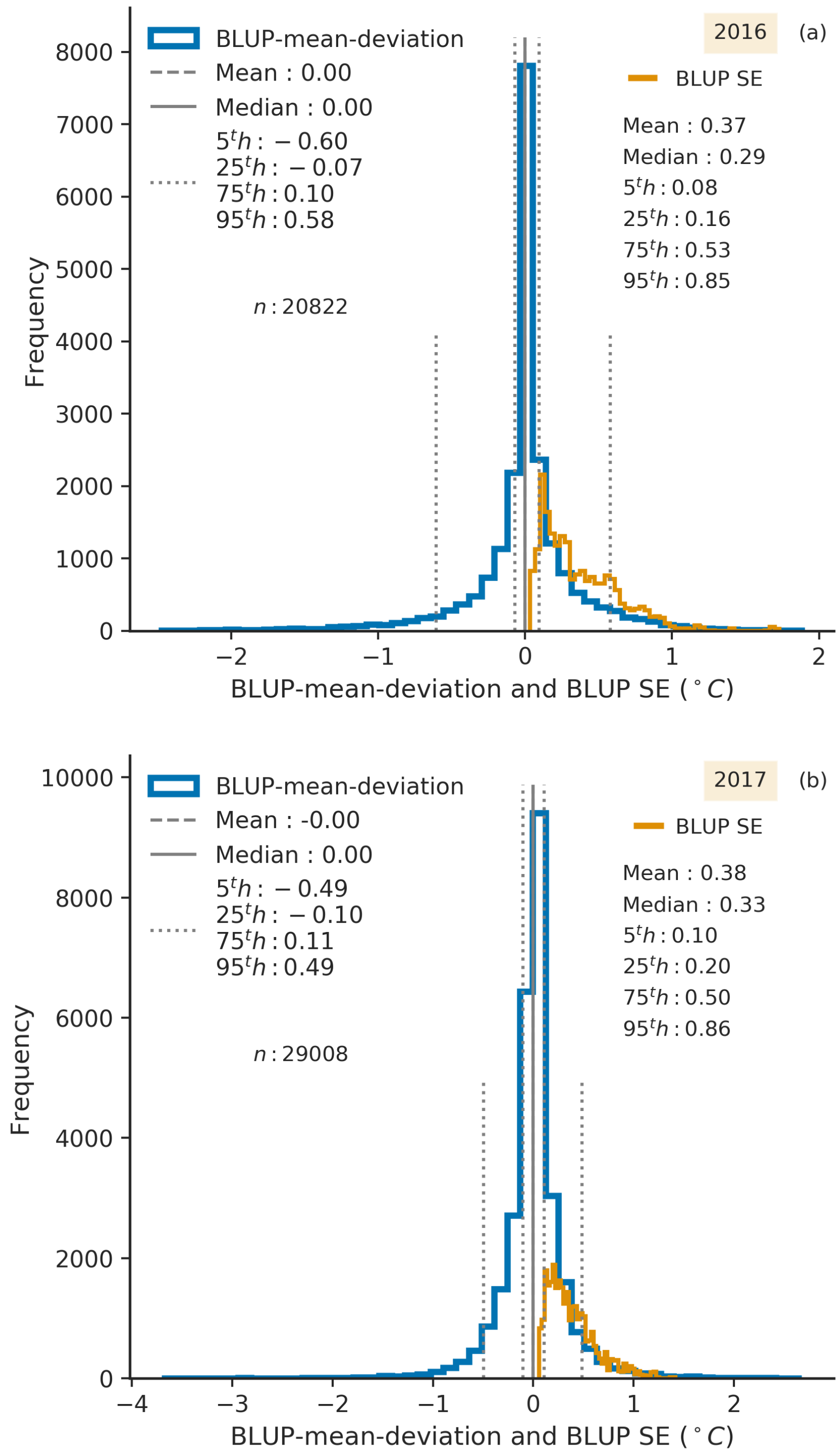

Figure S9. The best linear unbiased predictors of genotype effects (BLUPs) and standard errors (BLUP SEs) for ArduCrop canopy temperature (CT). BLUP-mean-deviation = BLUP-mean − BLUP. For a given date-by-time sampling event, BLUP-mean is the mean of all BLUPs. BLUP-mean-deviations and BLUP SEs for ArduCrop CT shown as frequency distributions for 2016 (a) and 2017 (b).

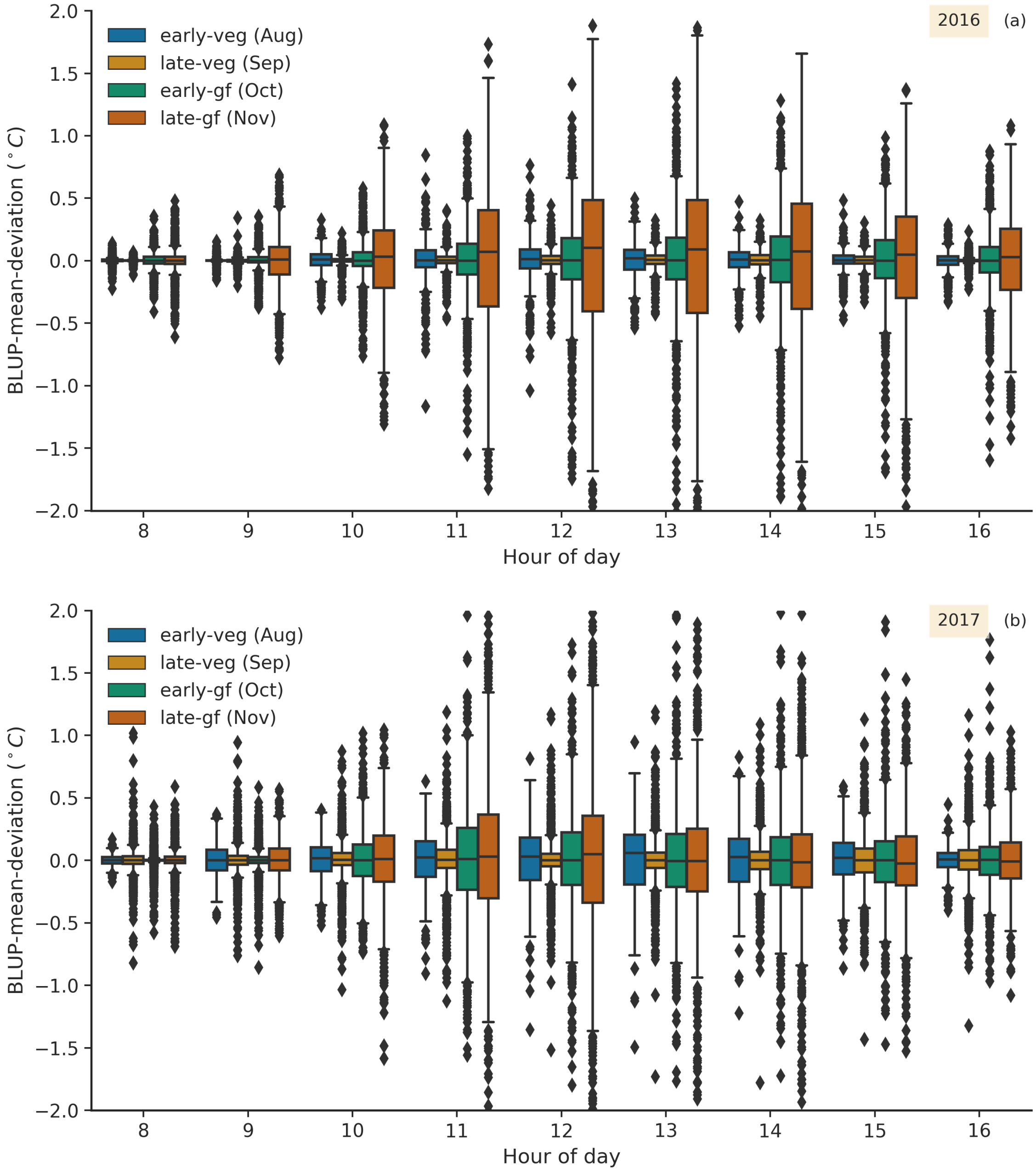

Figure S10. The best linear unbiased predictors of genotype effects (BLUPs) for ArduCrop canopy temperature (CT). BLUP-mean-deviation = BLUP-mean − BLUP. For a given date-by-time sampling event, BLUP-mean is the mean of all BLUPs. Box plots of BLUP-mean-deviations for each hour of the day grouped by growth stage for 2016 (a) and 2017 (b). Legend: early-veg, early vegetative; late-veg, late vegetative; early-gf, early grain-filling; late-gf, late grain-filling.

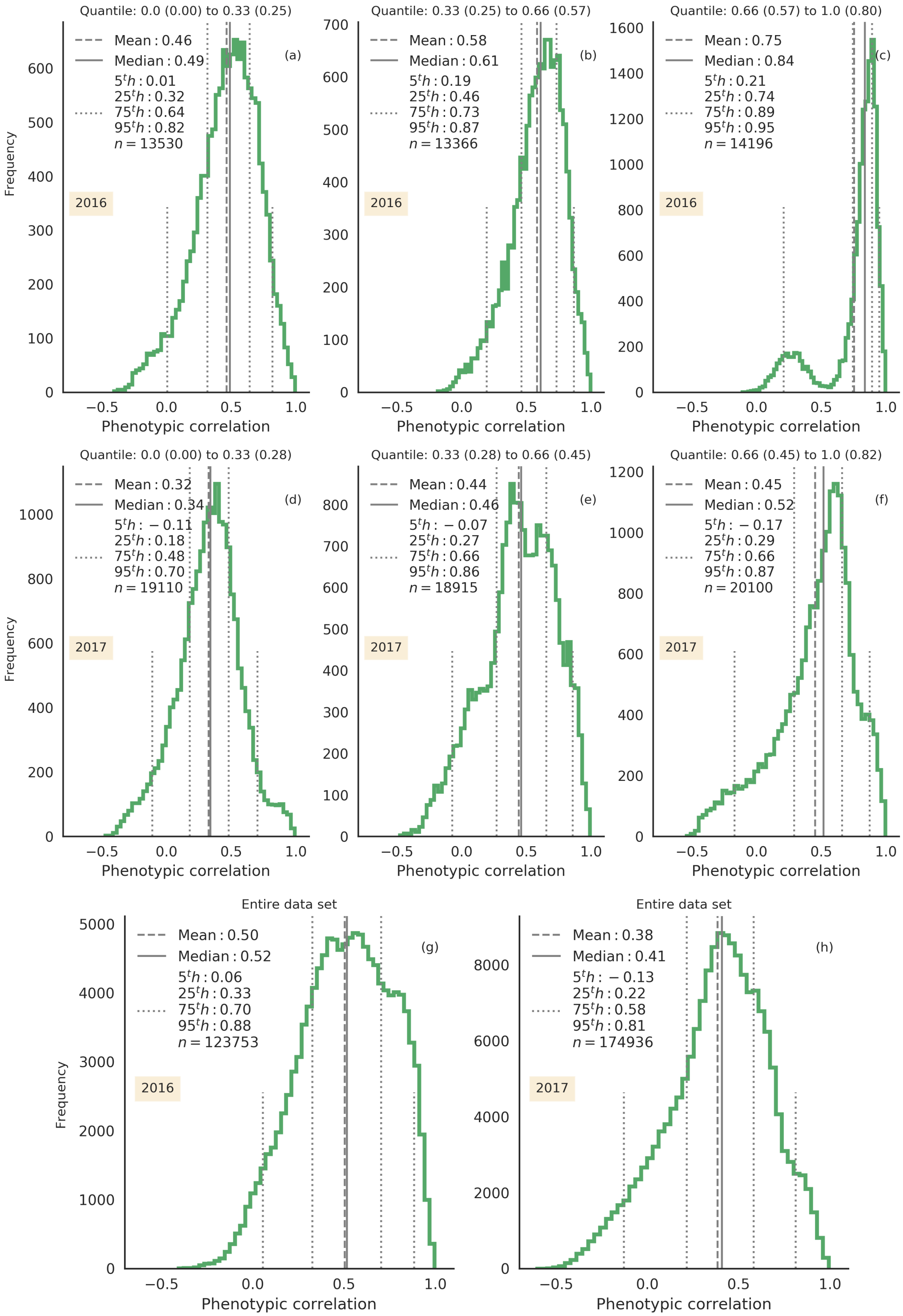

Figure S11. All possible pairwise phenotypic correlations between best linear unbiased predictors of genotype effects (BLUPs) for ArduCrop canopy temperature in 2016 and 2017, shown as frequency distributions for the following, arbitrarily chosen, event repeatability quantiles: 0.0 to 0.33 in 2016 (a) and 2017 (d); 0.33 to 0.66 in 2016 (b) and 2017 (e); 0.66 to 1.0 in 2016 (c) and 2017 (f). Values in parenthesis denote the respective quantile value of repeatability. Frequency distribution of phenotypic correlations between BLUPs for the entire data set in 2016 (g) and 2017 (h).

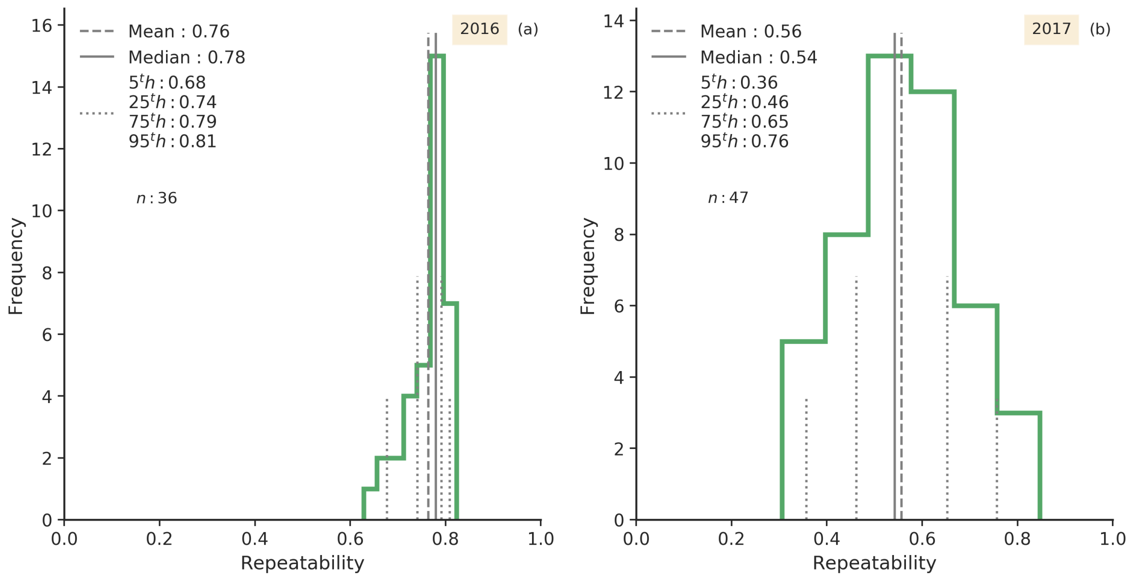

Figure S12. Repeatability estimates for airborne canopy temperature shown as frequency distributions for 2016 (a) and 2017 (b).

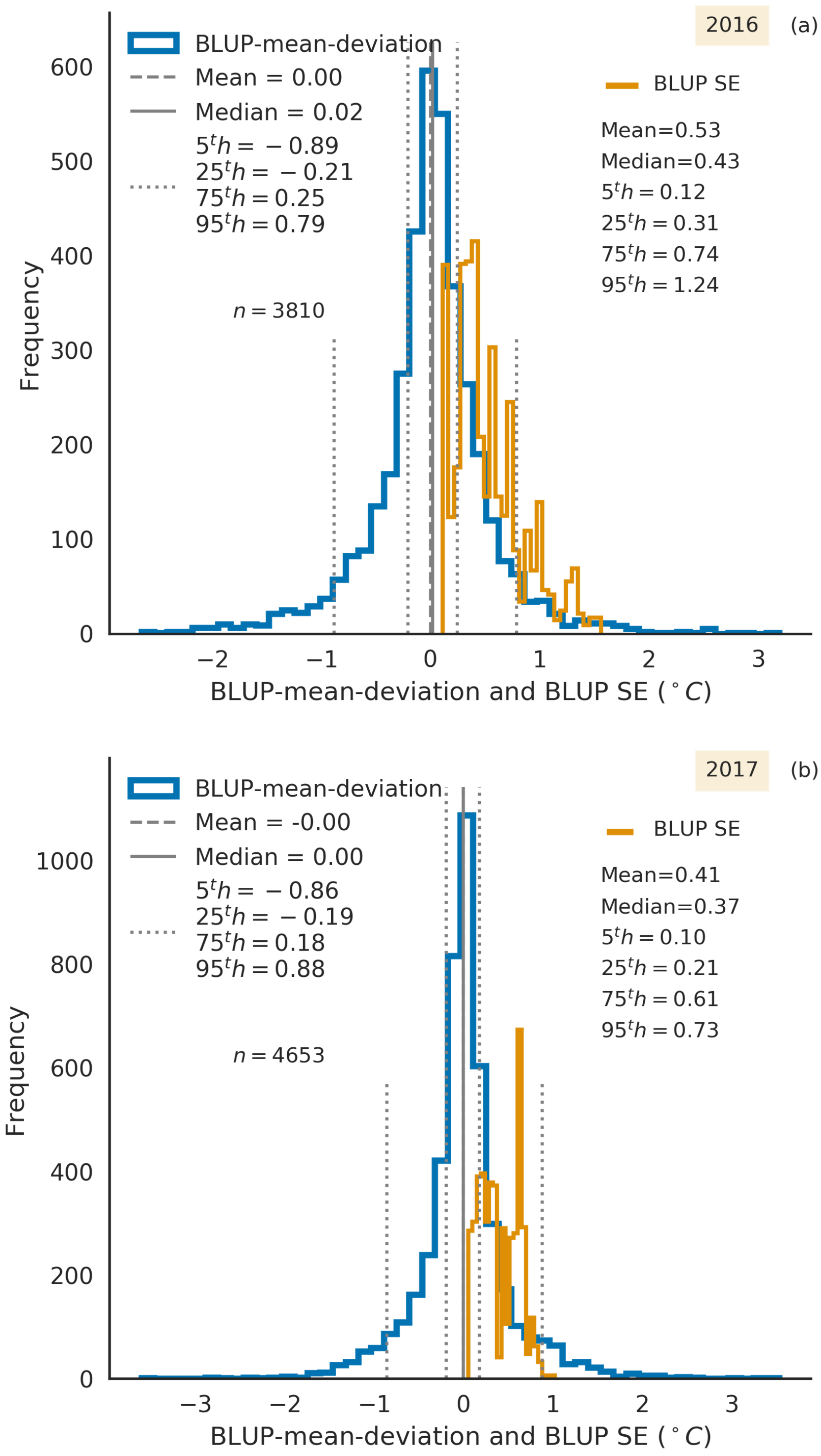

Figure S13. The best linear unbiased predictors of genotype effects (BLUPs) and standard errors (BLUP SEs) for airborne canopy temperature (CT). BLUP-mean-deviation = BLUP-mean − BLUP. Where for a given date-by-time sampling event, BLUP-mean is the mean of all BLUPs. BLUP-mean-deviations and BLUP SEs for airborne CT shown as frequency distributions for 2016 (a) and 2017 (b).

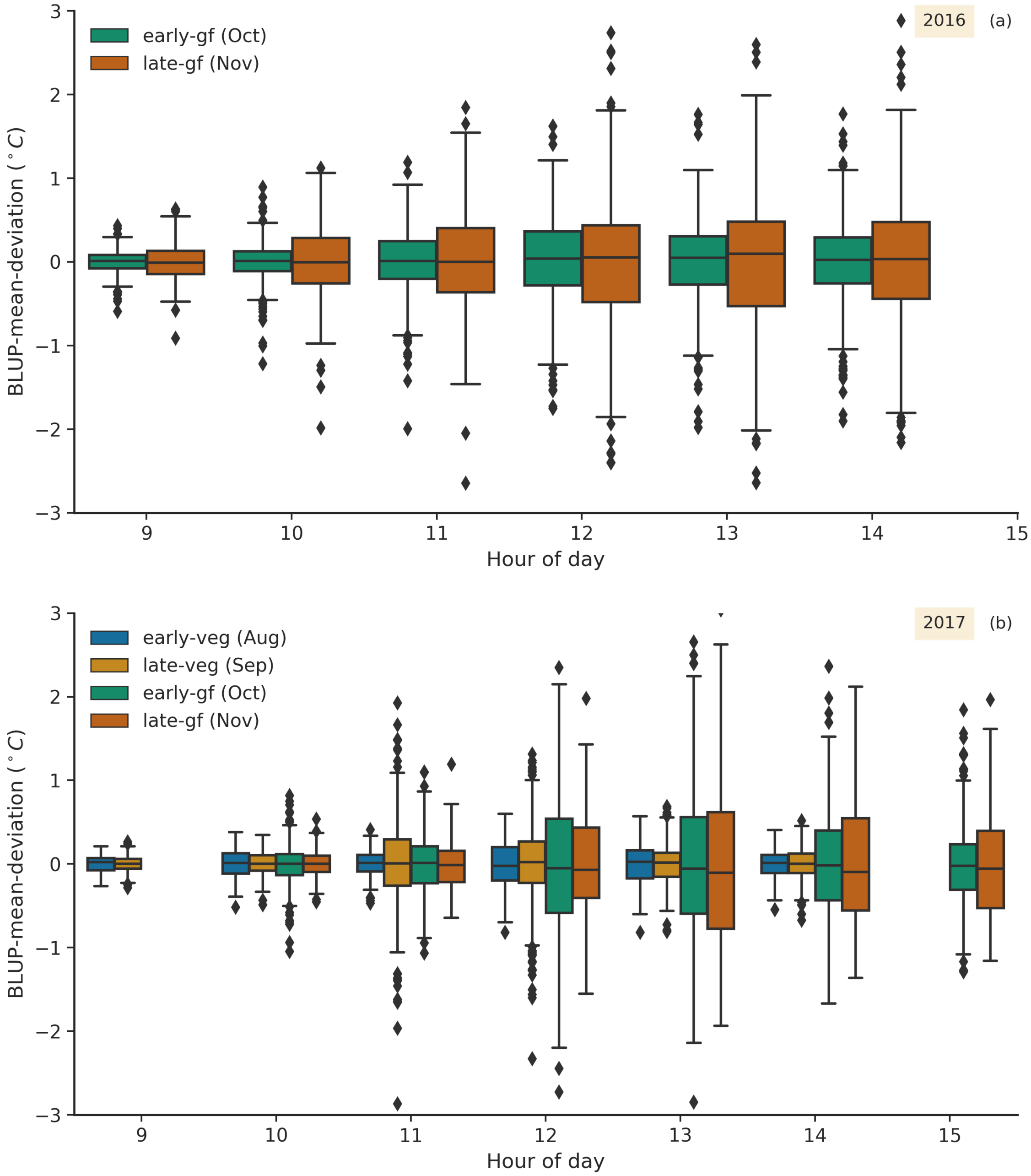

Figure S14. The best linear unbiased predictors of genotype effects (BLUPs) for airborne canopy temperature (CT). BLUP-mean-deviation = BLUP-mean − BLUP. Where for a given date-by-time sampling event, BLUP-mean is the mean of all BLUPs. BLUP-mean-deviations for airborne CT shown as box plots for each hour of the day grouped by growth stage for 2016 (a) and 2017 (b). Legend: early-veg, early vegetative; late-veg, late vegetative; early-gf, early grain-filling; late-gf, late grain-filling.

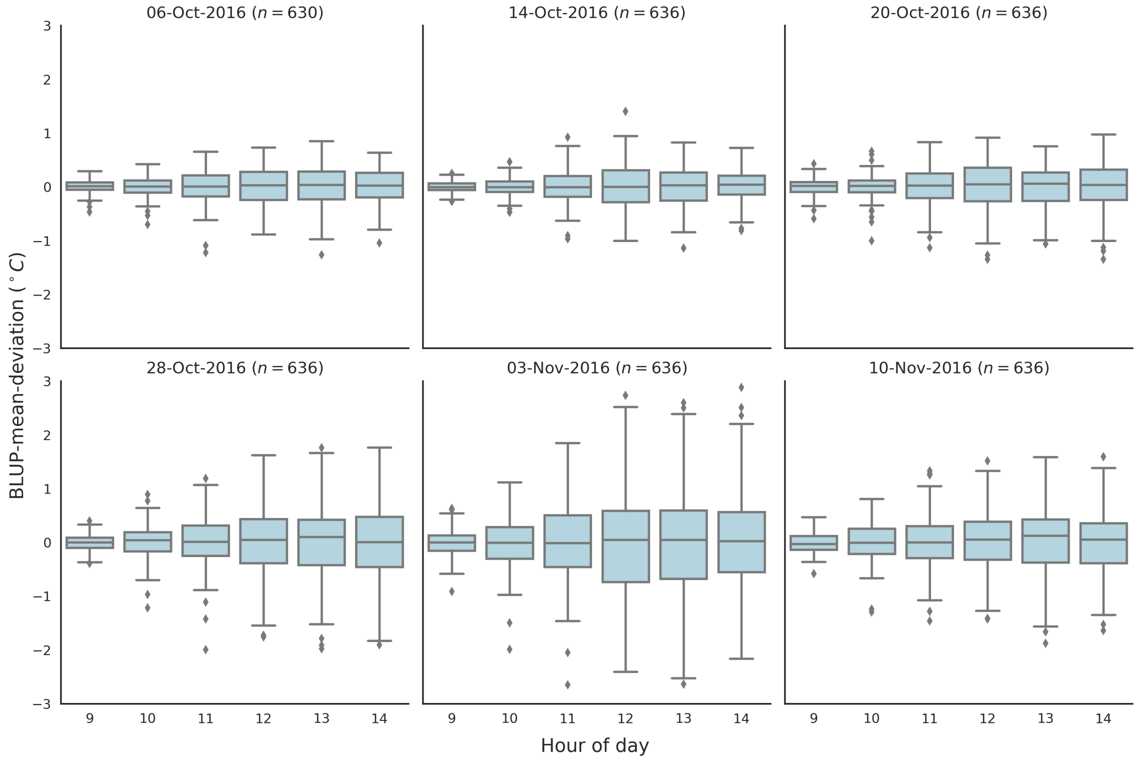

Figure S15. The best linear unbiased predictors of genotype effects (BLUPs) for airborne canopy temperature (CT) in 2016. BLUP-mean-deviation = BLUP-mean − BLUP. Where for a given date-by-time sampling event, BLUP-mean is the mean of all BLUPs. Box plots of BLUP-mean-deviations for each hour of the day grouped by day.

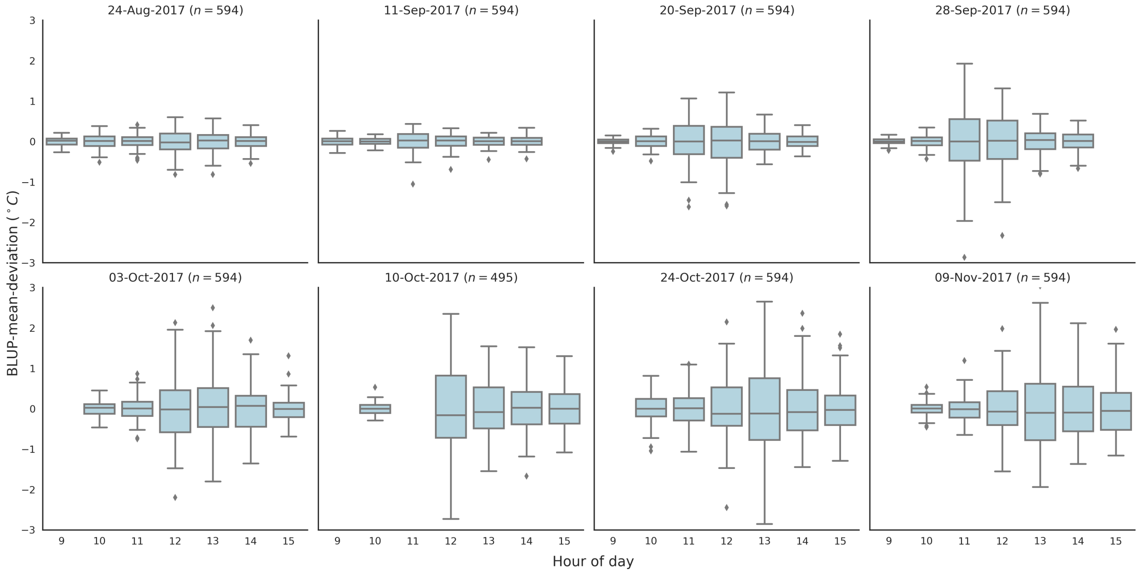

Figure S16. The best linear unbiased predictors of genotype effects (BLUPs) for airborne canopy temperature (CT) in 2017. BLUP-mean-deviation = BLUP-mean − BLUP. Where for a given date-by-time sampling event, BLUP-mean is the mean of all BLUPs. Box plots of BLUP-mean-deviations for each hour of the day grouped by day.

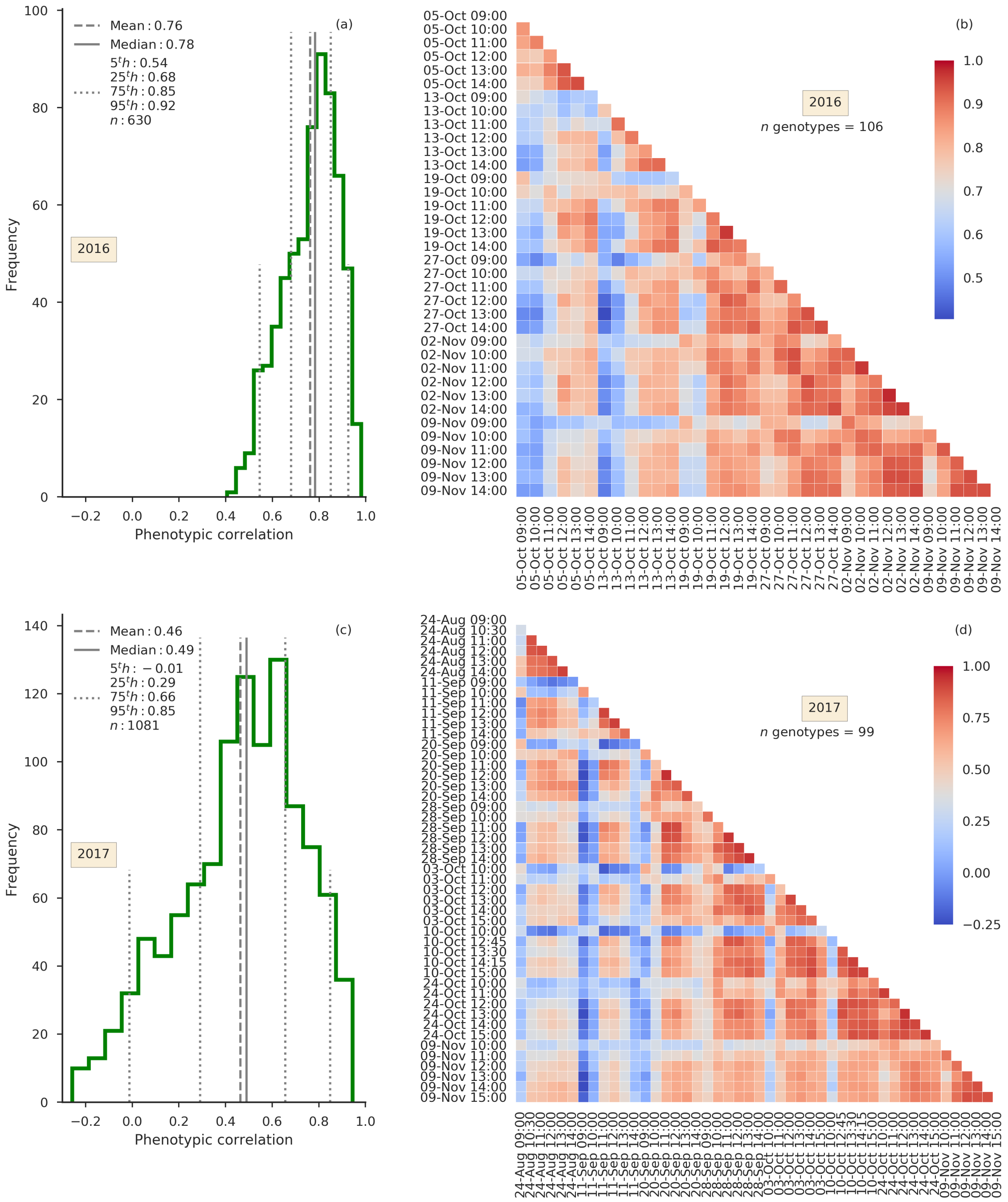

Figure S17. Phenotypic correlations between the best linear unbiased predictors of genotype effects for all airborne canopy temperature events in 2016 and 2017. Frequency distributions for 2016 (a) and 2017 (c). Heatmaps for 2016 (b) and 2017 (d).

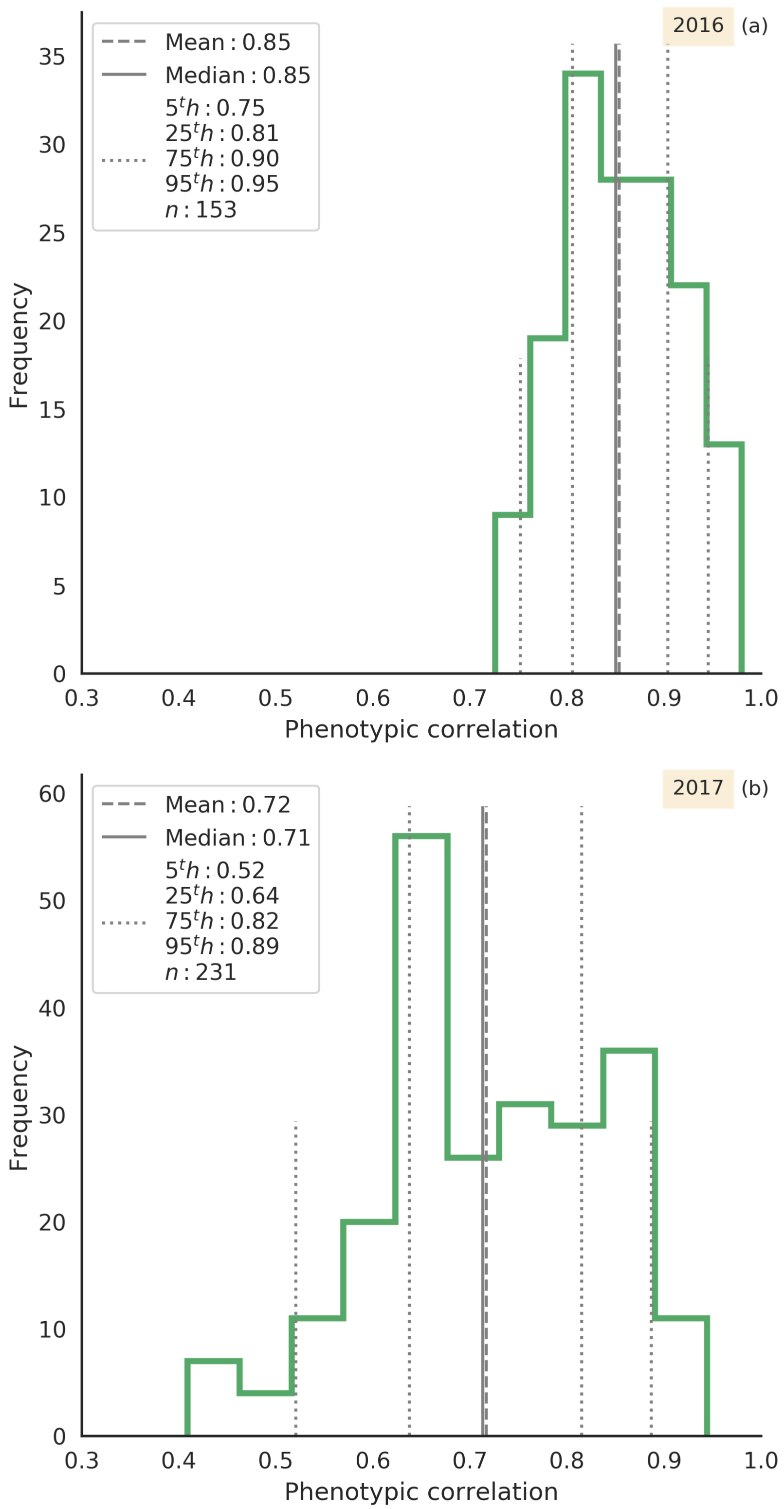

Figure S18. Frequency distributions of phenotypic correlations between the best linear unbiased predictors of genotype effects (BLUPs) for selected airborne canopy temperature events in 2016 (a) and 2017 (b). For 2016, on each day after (and including) 12:00. For 2017, for events on days after and including 20-Sept and after 12:00.

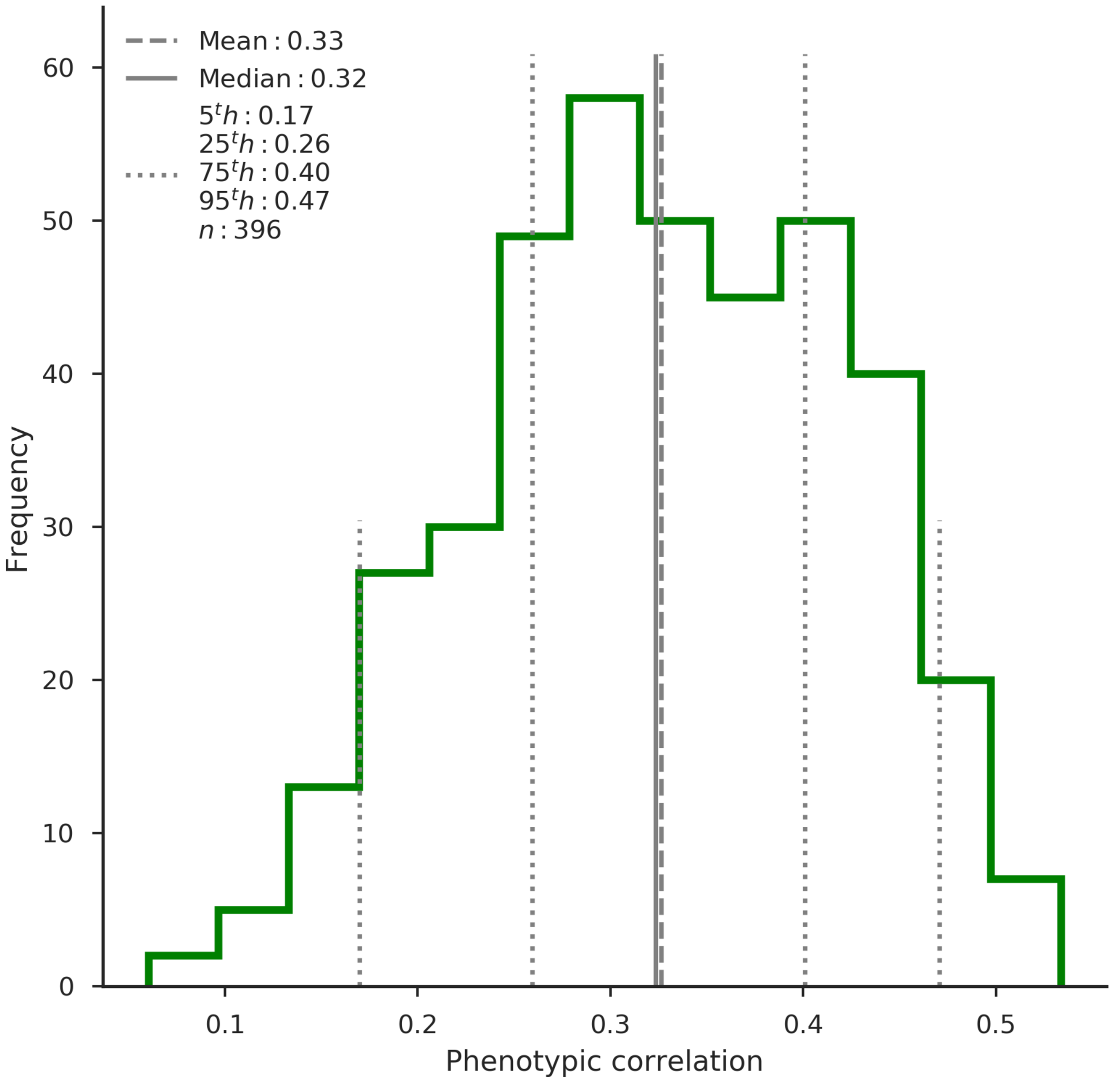

Figure S19. Frequency distribution of phenotypic correlations between the best linear unbiased predictors of genotype effects (BLUPs) (98 genotypes) for selected airborne canopy temperature events in 2016 and 2017. For 2016, on each day after (and including) 12:00. For 2017, for events on days after and including 20-Sept and after 12:00.

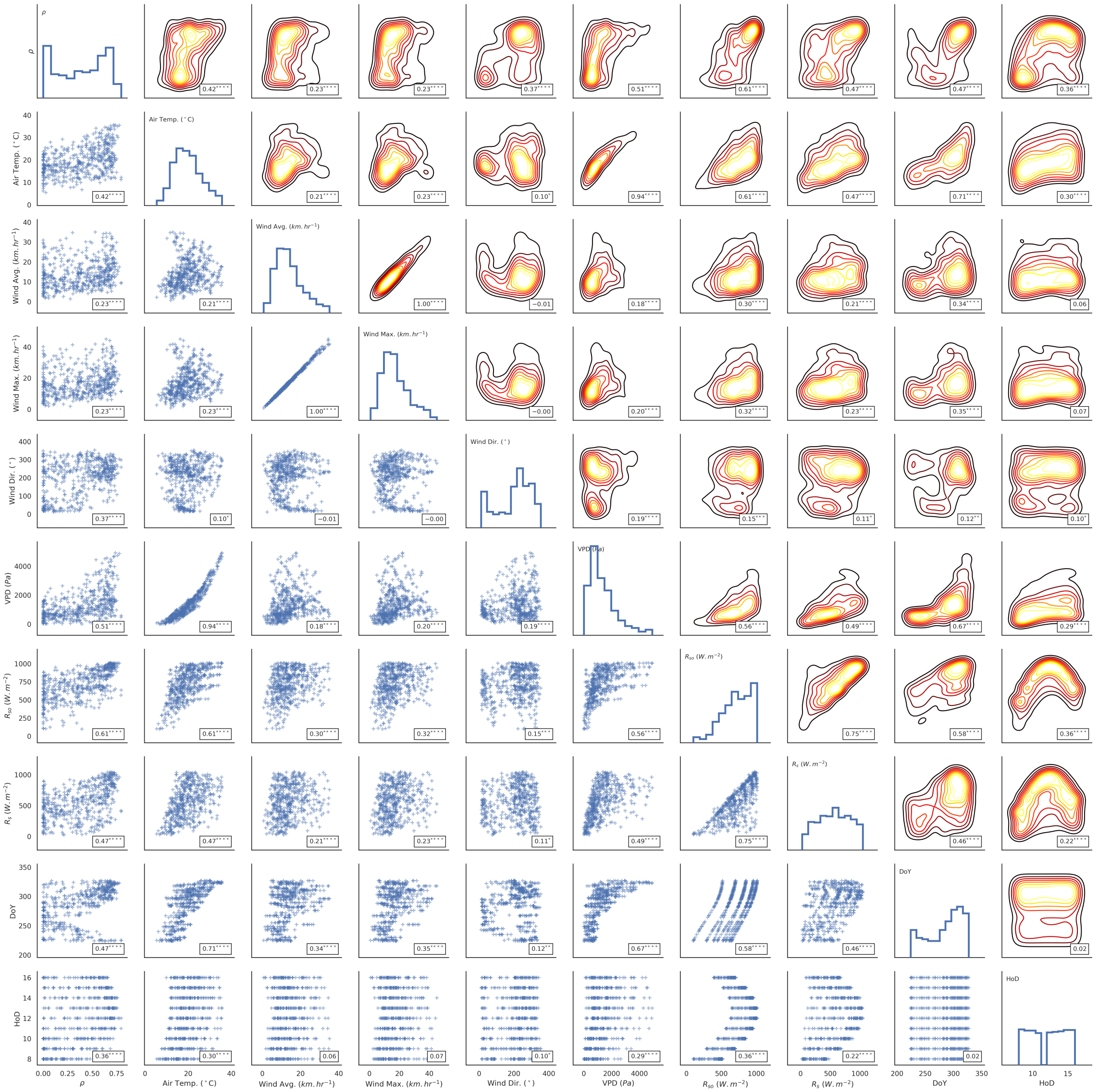

Figure S20. For ArduCrop canopy temperature in 2016, scatter plots (lower half) and kernel density estimates (upper half) between repeatability, ρ, weather variables, day-of-year (DoY) and hour-of-day (HoD) ($n = 954$). Pearson correlations are shown in the lower right for each plot and statistically significant associations are denoted: ****, $P < 0.0001$; ***, $P < 0.001$; **, $P < 0.01$; *, $P < 0.05$. VPD is vapour pressure deficit (Pa), R_{so} ($MJ.m^{-2}$) is the calculated clear-sky solar radiation and R_s ($MJ.m^{-2}$) is the measured solar radiation at Griffith NSW (ca. 60 km north-west from the experiment site). The histogram plots show the frequency distribution for each respective variable.

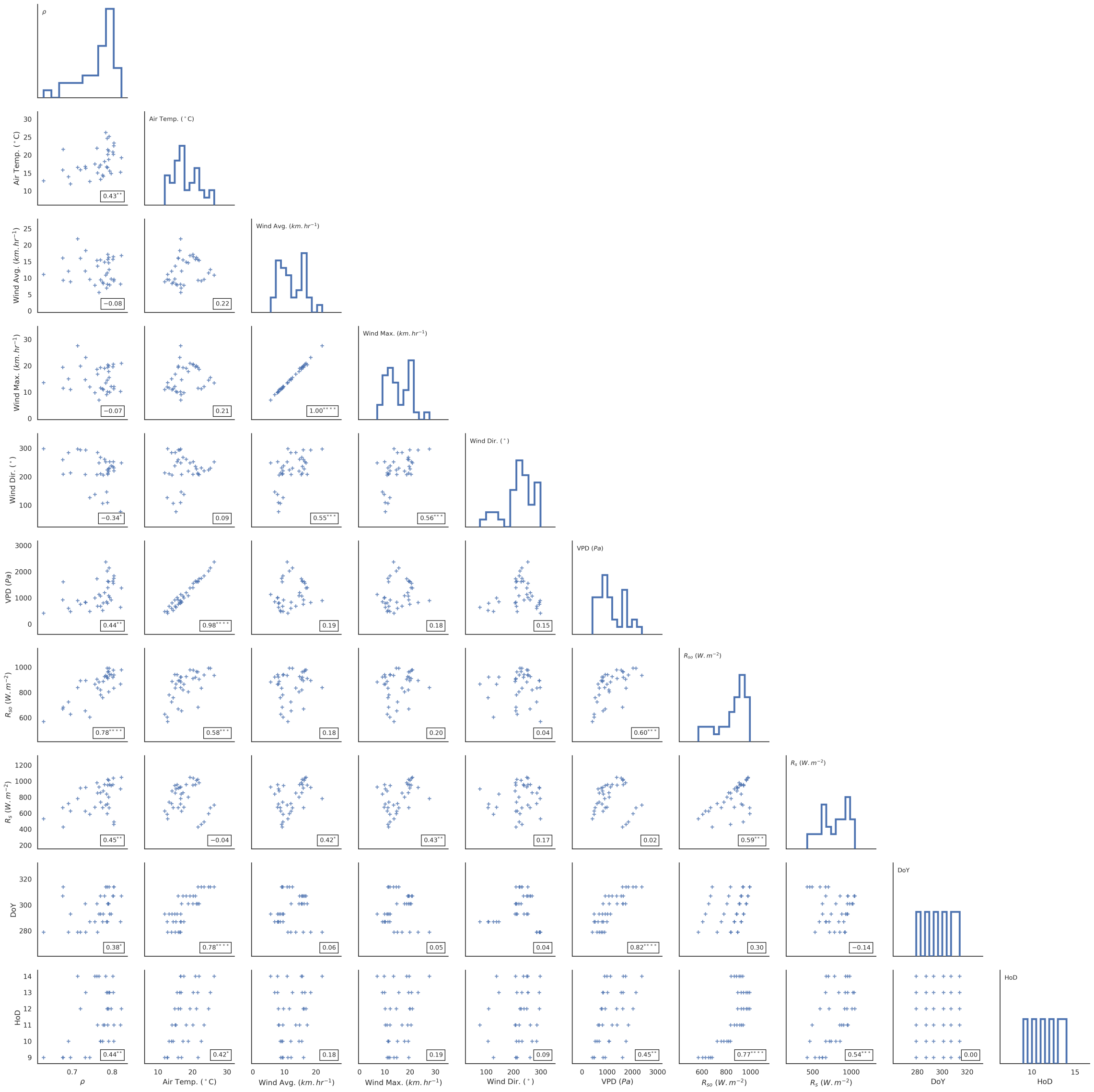

Figure S21. For airborne canopy temperature in 2016, scatter plots between repeatability, ρ, weather variables, day-of-year (DoY) and hour-of-day (HoD) ($n = 36$). Pearson correlations are shown in the lower right for each plot and statistically significant associations are denoted: ****, $P < 0.0001$; ***, $P < 0.001$; **, $P < 0.01$; *, $P < 0.05$. VPD is vapour pressure deficit (Pa), R_{so} ($MJ.m^{-2}$) is the calculated clear-sky solar radiation and R_s ($MJ.m^{-2}$) is the measured solar radiation at Griffith NSW (ca. 60 km north-west from the experiment site). The histogram plots show the frequency distribution for each respective variable.

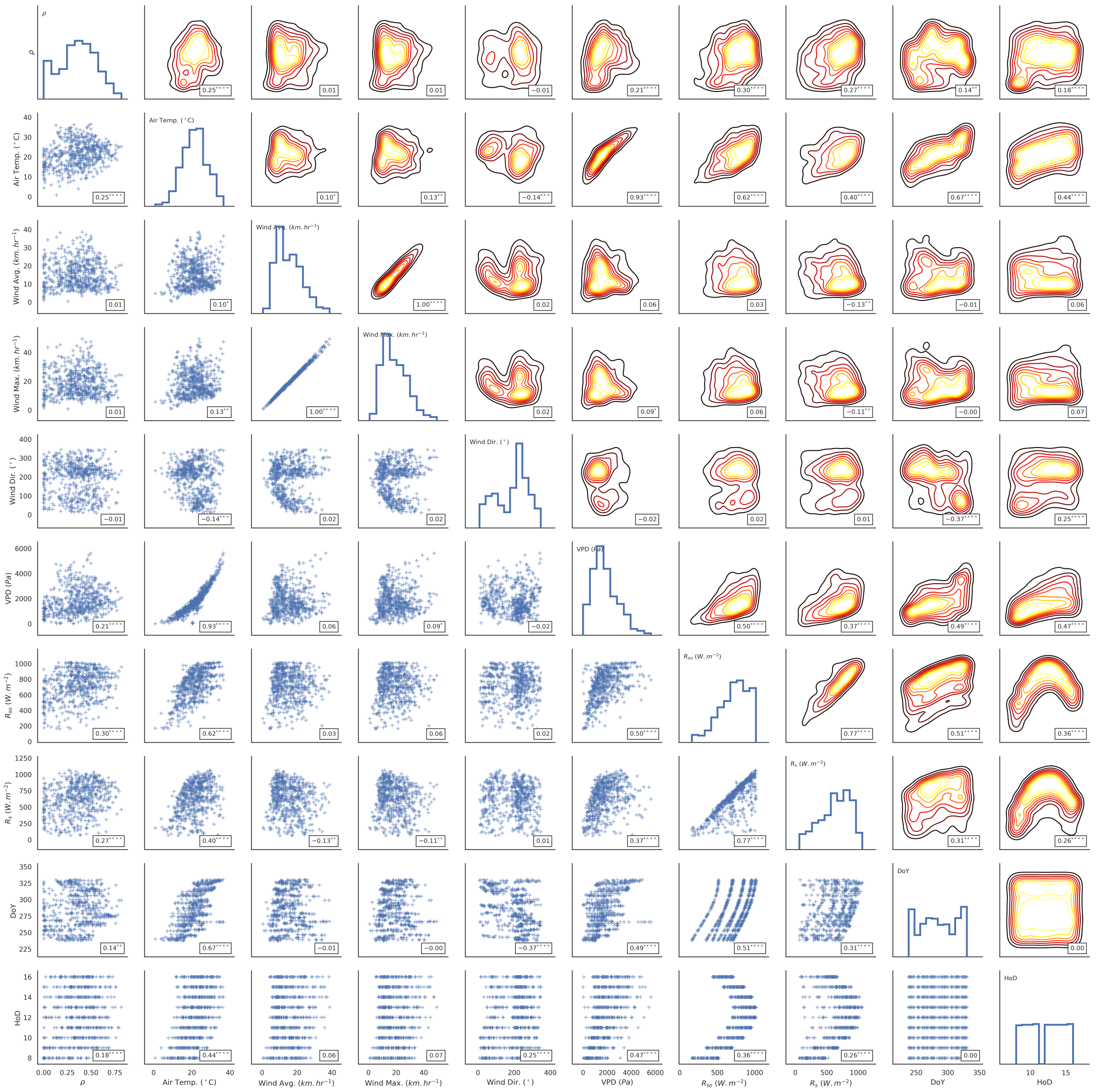

Figure S22. For ArduCrop canopy temperature in 2017, scatter plots (lower half) and kernel density estimates (upper half) between repeatability, ρ, weather variables, day-of-year (DoY) and hour-of-day (HoD) ($n = 799$). Pearson correlations are shown in the lower right for each plot and statistically significant associations are denoted: ****, $P < 0.0001$; ***, $P < 0.001$; **, $P < 0.01$; *, $P < 0.05$. VPD is vapour pressure deficit (Pa), R_{so} ($MJ.m^{-2}$) is the calculated clear-sky solar radiation and R_s ($MJ.m^{-2}$) is the measured solar radiation at Griffith NSW (ca. 60 km north-west from the experiment site). The histogram plots show the frequency distribution for each respective variable.

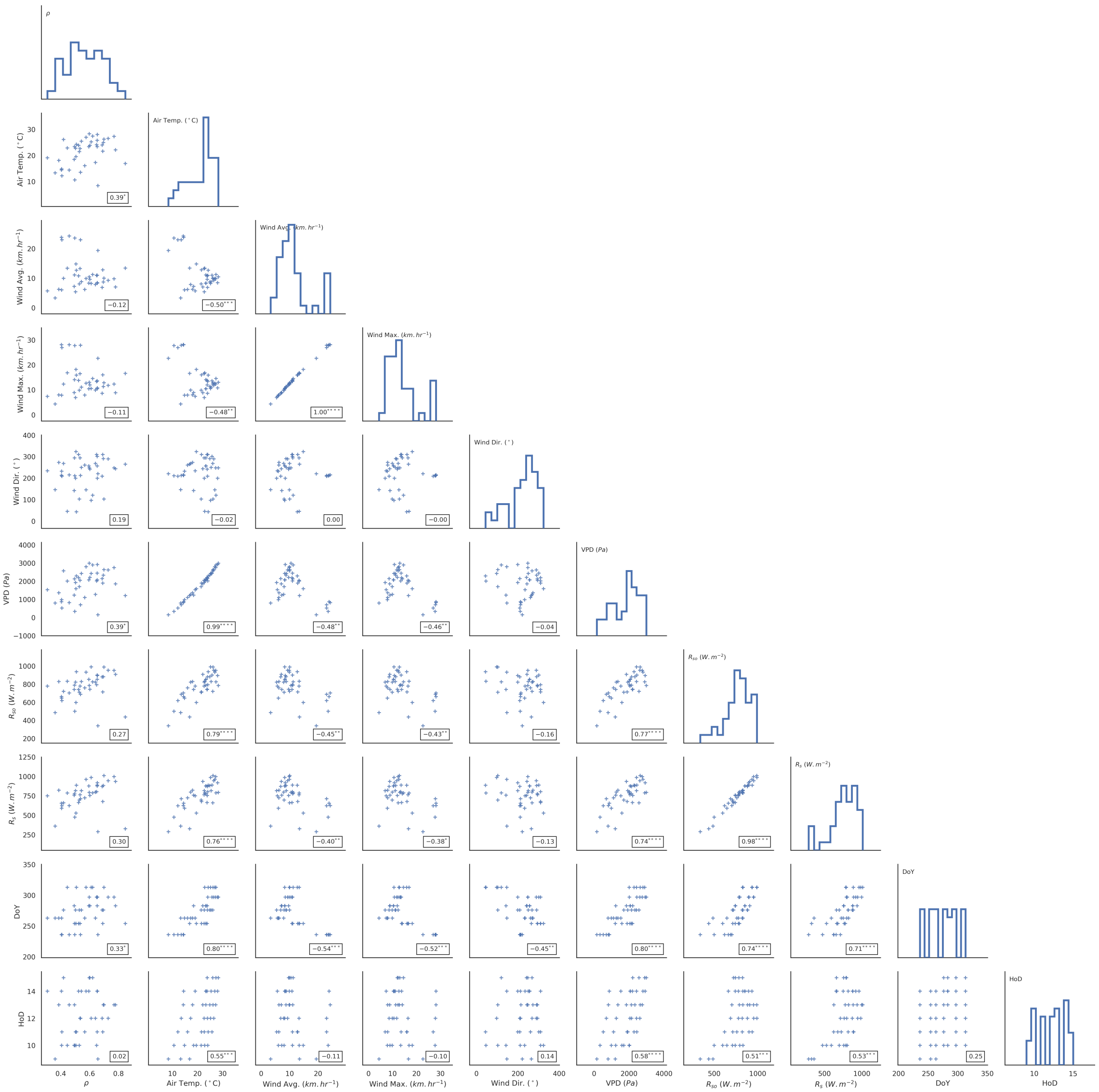

Figure S23. For airborne canopy temperature in 2017, scatter plots between repeatability, ρ, weather variables, day-of-year (DoY) and hour-of-day (HoD) ($n = 47$). Pearson correlations are shown in the lower right for each plot and statistically significant associations are denoted: ****, $P < 0.0001$; ***, $P < 0.001$; **, $P < 0.01$; *, $P < 0.05$. VPD is vapour pressure deficit (Pa), R_{so} ($MJ.m^{-2}$) is the calculated clear-sky solar radiation and R_s ($MJ.m^{-2}$) is the measured solar radiation at Griffith NSW (ca. 60 km north-west from the experiment site). The histogram plots show the frequency distribution for each respective variable.

2 TABLES

Table S1. From Figure S2, summary of linear regression parameters for each of the 29 plots that comprised two paired ArduCrop sensors in 2016 (i.e. internal ArduCrop replication denoted ArduCrop 1 and ArduCrop 2). Results include the slope, intercept, coefficient of determination (R^2), root mean square error (RMSE), normalised RMSE (NRMSE) and the number of values (n).

	Slope	Intercept	R^2	RMSE	NRMSE	n
Mean	0.99	0.17	0.98	1.10	0.05	427
SD	0.05	0.69	0.01	0.40	0.02	73
Min	0.91	-1.32	0.96	0.55	0.03	180
25%	0.96	-0.26	0.98	0.73	0.04	415
50%	0.99	0.18	0.99	1.04	0.05	417
75%	1.03	0.58	0.99	1.32	0.06	496
Max	1.11	1.31	1.00	1.89	0.09	497

Table S2. Ordinary least squares model results for canopy temperature (CT) repeatability (response variable) and VPD, day of year (DoY) and hour of day (HoD). DoY and HoD are key determinants of R_{so} and were used here instead of R_{so} to test for their significance. Statistically significant P-values are denoted: ****, $P < 0.0001$; ***, $P < 0.001$; **, $P < 0.01$; *, $P < 0.05$; ns, not significant. Scatter plot associations between CT repeatability estimates and corresponding hourly weather data are shown in Figures S20, S21, S22 and S23 for 2016 ArduCrop, 2016 airborne, 2017 ArduCrop and 2017 airborne, respectively.

	Variables			P-values			R^2
				VPD	DoY	HoD	
2016 ArduCrop CT	DoY				****		0.753
	HoD					****	0.751
	DoY	HoD			****	****	0.763
	VPD	DoY	HoD	****	ns	****	0.804
2016 Airborne CT	DoY				****		0.997
	HoD					****	0.983
	DoY	HoD			****	**	0.998
	VPD	DoY	HoD	ns	****	***	0.998
2017 ArduCrop CT	DoY				****		0.784
	HoD					****	0.777
	DoY	HoD			****	****	0.792
	VPD	DoY	HoD	*	****	***	0.793
2017 Airborne CT	DoY				****		0.960
	HoD					****	0.936
	DoY	HoD			****	ns	0.961
	VPD	DoY	HoD	ns	****	ns	0.961
